# The expression of the acarbose biosynthesis gene cluster in *Actinoplanes* sp. SE50/110 is dependent on the growth phase

**DOI:** 10.1186/s12864-020-07194-6

**Published:** 2020-11-23

**Authors:** Julian Droste, Vera Ortseifen, Lena Schaffert, Marcus Persicke, Susanne Schneiker-Bekel, Alfred Pühler, Jörn Kalinowski

**Affiliations:** 1 Microbial Genomics and Biotechnology, Center for Biotechnology, Bielefeld University, Sequenz 1, Bielefeld, 33615 Germany; 2 Senior Research Group in Genome Research of Industrial Microorganisms, Center for Biotechnology, Bielefeld University, Sequenz 1, 33615 Bielefeld, Germany

**Keywords:** *Actinoplanes*, Acarbose, Transcriptomic, Proteomic, Expression dynamics, Co-regulation

## Abstract

**Background:**

*Actinoplanes* sp. SE50/110 is the natural producer of the diabetes mellitus drug acarbose, which is highly produced during the growth phase and ceases during the stationary phase. In previous works, the growth-dependency of acarbose formation was assumed to be caused by a decreasing transcription of the acarbose biosynthesis genes during transition and stationary growth phase.

**Results:**

In this study, transcriptomic data using RNA-seq and state-of-the-art proteomic data from seven time points of controlled bioreactor cultivations were used to analyze expression dynamics during growth of *Actinoplanes* sp. SE50/110. A hierarchical cluster analysis revealed co-regulated genes, which display similar transcription dynamics over the cultivation time. Aside from an expected metabolic switch from primary to secondary metabolism during transition phase, we observed a continuously decreasing transcript abundance of all acarbose biosynthetic genes from the early growth phase until stationary phase, with the strongest decrease for the monocistronically transcribed genes *acbA, acbB, acbD* and *acbE*. Our data confirm a similar trend for *acb* gene transcription and acarbose formation rate.

Surprisingly, the proteome dynamics does not follow the respective transcription for all *acb* genes. This suggests different protein stabilities or post-transcriptional regulation of the Acb proteins, which in turn could indicate bottlenecks in the acarbose biosynthesis. Furthermore, several genes are co-expressed with the *acb* gene cluster over the course of the cultivation, including eleven transcriptional regulators (e.g. ACSP50_0424), two sigma factors (ACSP50_0644, ACSP50_6006) and further genes, which have not previously been in focus of acarbose research in *Actinoplanes* sp. SE50/110.

**Conclusion:**

In conclusion, we have demonstrated, that a genome wide transcriptome and proteome analysis in a high temporal resolution is well suited to study the acarbose biosynthesis and the transcriptional and post-transcriptional regulation thereof.

**Supplementary Information:**

**Supplementary information** accompanies this paper at 10.1186/s12864-020-07194-6.

## Background

*Actinoplanes* sp. SE50/110 is a Gram-positive, aerobic bacterium belonging to the genus of *Actinoplanes*, within the family *Micromonosporaceae* [1, 2]. Members of the genus *Actinoplanes* can form sporangia, that contain motile spores, and typically grow in branched hyphae [1, 3]. *Actinoplanes* spp. are characterized by genomes with high G + C contents of 69–73% [1, 3]. Several species are known for their potential to produce a variety of secondary metabolites, like antibiotics [4, 5]. Among them are more than 120 antibiotics, like actaplanin [6], teicoplanin [7], friulimicins [8] and ramoplanin [9]. *Actinoplanes* sp. SE50 strains are of special interest because of their ability to produce the pseudotetrasaccharide acarbose, which has an inhibitory effect on alpha-glucosidases and is therefore of special interest for pharmaceutical applications [10]. Due to its inhibitory effect, acarbose is used for the treatment of diabetes mellitus. The inhibition of the intestinal alpha-glucosidases decelerates the degradation of long-chain carbons and thus leads to a retarded resorption of monosaccharides into the blood system [11–13]. By this, the postprandial blood and serum sugar glucose is reduced, which is a risk factor for developing secondary complications, like cardiovascular diseases, diabetical retinopathies and diabetic food syndrome [14].

The strain *Actinoplanes* sp. SE50/110 is the best-studied acarbose producer and a high quality genome sequence is known [15]. Several biochemical studies of the enzymes of the acarbose biosynthesis (*acb*) gene cluster and genomic as well as proteomic studies were carried out to propose pathways for the biosynthesis of acarbose [13, 16–19]. Recently, tools for genome editing based on CRISPR/Cas9 [20], an overexpression system using different promoter strengths [21] and a protocol for conjugational plasmid transfer [22] were developed for *Actinoplanes* sp. SE50/110, which will further promote the acarbose research in this strain.

The transcriptional organization of the *acb* gene cluster, including transcription start sites, promoter elements and operon organization was recently elucidated [15]. The *acb* gene cluster in *Actinoplanes* sp. SE50/110 lacks genes coding for transcription factors. This is in contrast to the acarbose biosynthetic gene clusters of *Streptomyces* spp. [23, 24]. Only one study concerning a transcription factor involved in acarbose biosynthesis and its binding sites is known [25]. Since it is known that the formation of acarbose correlates with the course of cell growth and no acarbose is produced in the stationary phase [26, 27], the expression dynamics of genes involved in the acarbose biosynthesis of *Actinoplanes* sp. SE50/110 was examined in this study. Bioreactor cultivations were conducted to maintain controlled cultivation conditions and both transcriptomic and proteomic analyses were used in a high temporal resolution to study whole genome expression dynamics during the course of cell growth. A hierarchical cluster analysis of the data was performed to elucidate co-expressed genes. Finally, acarbose biosynthesis (*acb*) genes were analyzed in detail regarding their respective transcript and protein dynamics. Furthermore, genes co-expressed to the *acb* gene cluster were elucidated.

## Results and discussion

### Acarbose production of *Actinoplanes* sp. SE50/110 steadily decreases during the growth phase and almost ceases in stationary phase

In this study, the changes of acarbose production during the growth of *Actinoplanes* sp. SE50/110 were analyzed. Therefore, bioreactor cultivations were used to achieve controlled cultivation parameters. *Actinoplanes* sp. SE50/110 was cultivated in maltose minimal medium in three biological replicates. Spores were generated for inoculation by first growing *Actinoplanes* sp. SE50/110 in NBS complex medium and afterward plating the cells on SFM agar plates to generate spores, which in turn served as inoculum. Samples were taken at regular intervals to monitor the course of growth and acarbose formation.

**Fig. 1:**
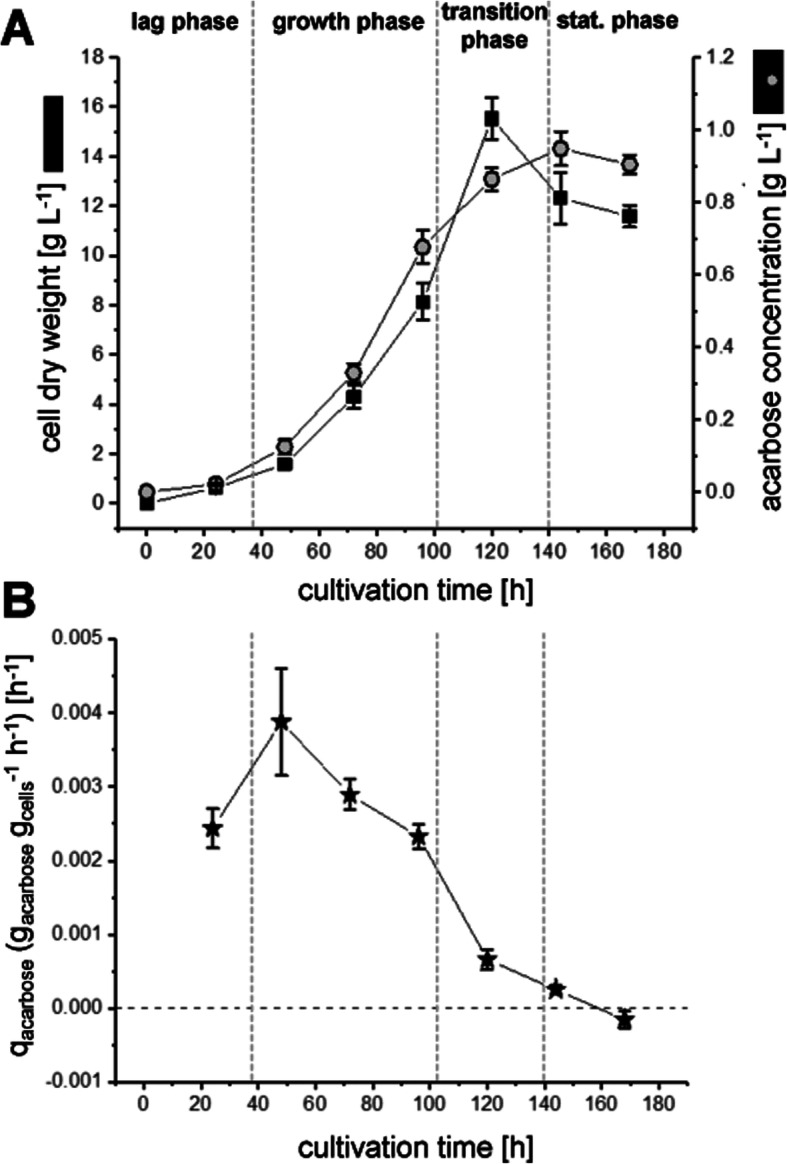
Characterization of growth and acarbose production of *Actinoplanes* sp. SE50/110 in controlled fermenter conditions. Different growth phases (lag, growth, transition and stationary phase) were indicated by vertical dashed lines. **a** Cell dry weight (black boxes) and acarbose concentration (grey circles) over the cultivation course. Plotted are the means and standard deviations of three biological replicates, each of which were measured in three technical replicates. **b** Specific product formation rates (q_Acarbose_) defined as produced acarbose normalized on the mean cell dry weight and cultivation time difference

Within the controlled conditions of reactor cultivations, a correlation between acarbose formation and the course of biomass production over time was shown (Fig.1a) as it was observed in previous studies [26]. Acarbose was produced, starting in the lag phase (24.0 h) and continuing during growth (47.8, 72.3, 96.5 h), until the cultivations reached the transition phase (120.0 to 144.3 h). The acarbose concentration in the supernatant remains almost constant during the stationary phase (144.3 and 168.0 h). The specific product formation rate, defined as produced acarbose normalized to the mean cell dry weight and to cultivation time, increased during the first 48 h and then decreased steadily (Fig. 1b). The specific product formation is a direct indicator for acarbose production of the mycelial growing strain during a defined period and is not biased by hitherto formed acarbose. The findings of an acarbose production by *Actinoplanes* sp. SE50/110 in a growth-dependent manner is in good accordance to shake flask cultivations reported in the literature [25, 26]. For further analyses of the growth dependency of acarbose formation transcriptome and proteome dynamics were examined over the whole fermentation process.

### Analysis of whole transcriptome data of *Actinoplanes* sp. SE50/110

#### Processing and filtering of transcriptomic data

Whole transcriptome analysis using RNA-seq was subsequently carried out, in which seven time points in three biological replicates were compared to RNA pooled from all analyzed time points for each replicate. Thereby, a normalized analysis over the entire course of cultivation is possible, minimizing technical and biological variances. Consequently, a relative transcript abundance of one and a log_2_(fold change) of zero correspond to the average amount of transcript over all time points. For 8364 of all 8402 annotated features (99.5%), reads could be found for all analyzed time points. A principal component analysis (PCA) was performed to determine the differences of each time point to the pooled sample (Supplementary Figure 1). For cluster analyses genes were ruled out, if the transcription shows no significant difference (p_adj_-value > 0.05) at all time points compared to the mean value of the respective transcript. This filtering results in 6,770 genes with a significant different transcription for at least one time point. A schematic overview of processing and filtering steps can be found in Supplementary Figure 2.

#### Overview of temporal transcriptome dynamics

**Fig. 2:**
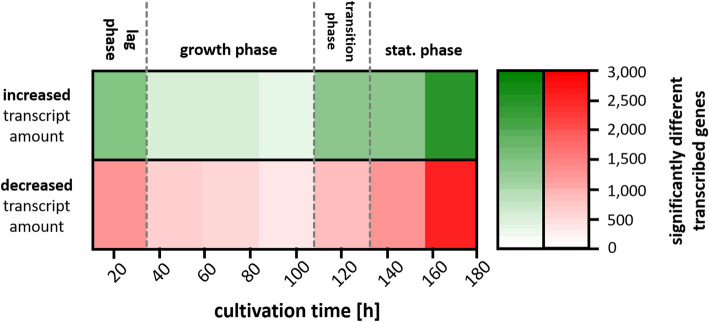
Overview about transcriptome dynamics in *Actinoplanes* sp. SE50/110. Numbefvficantly (p_adj_ -value < 0.05) increased (green) and decreased (red) transcript abundances during cultivation at the given time points. Growth phases are indicated with dashed lines

To gain a first overview, the number of genes was evaluated for which significantly increased or decreased transcript amounts were measured (p_adj_-value < 0.05) (Fig. 2). The highest number of genes with a significant difference in transcript amount compared to the respective average amount over the whole cultivation time was observed during the lag phase (24 h) and the late stationary phase (168 h). The transcript amount was significantly increased for 1421 (17.0%) genes and decreased for 1246 (14.9%) genes in the lag phase (24 h). In the late stationary phase 2491 (29.8%) of all genes show an increased and 2531 (30.3%) a decreased transcript amount. The minimal differences regarding transcription was observed in the mid growth phase (96.5 h).

Most genes are transcribed during filamentous growth and show their mean transcription level in the mid growth phase. The observed trend of differences in transcription is in good accordance to the expectation as a minimal number of differentially transcribed genes is expected during filamentous growth [28]. In contrast to that, the greatest difference regarding transcription was observed for the late stationary phase (168 h). This could be a hint for a typical switch from primary to secondary metabolism [28, 29].

However, it should be noted that this first global analysis highlights only genes with significantly differential transcript amounts at single time points and ignores trends in temporal transcriptome dynamics of single genes. To analyze these trends and identify co-regulated genes on a transcriptional level, a hierarchical cluster analysis was implemented, as it is described in material and methods section.

#### Identification of operon structures by combining whole transcriptome data sets of different time points

Two or more genes, that are transcribed from a single promoter, form an operon. The analysis of the operon structure of *Actinoplanes* sp. SE50/110 is an important step to investigate the co-regulation of single genes and large operons. The operon detection was performed using the software ReadXplorer [30]. The data of all 21 RNA-seq experiments were combined to increase the number of reads in regions with low coverage. The identified primary operons were checked for experimental validation using the TSS determined from the data of sequenced 5′-end enriched libraries from [15]. If an operon has an assigned TSS, it is experimentally validated. If not, it was specified as predicted operon. The class of sub-operons consists of operons which show a TSS for a posterior gene in a primary operon. All other genes, which could not be connected to an operon, were assigned to be monocistronically transcribed.

Under the studied conditions 1029 primary operons containing 2751 genes could be detected by combining the whole transcriptome data sets of all analyzed time points (Table 1, Supplementary Fig 2).

**Table 1:** Number of monocistronic genes, primary operons and sub-operons of *Actinoplanes* sp. SE50/110 obtained from operon analysis using ReadXplorer [30] obtained from RNA-seq data of this study

Genes per transcript	Primary operons	Sub-operons	Monocistronic genes
1	–	604	4757
2	689	63	–
3	181	32	–
4	95	17	–
≥ 5	64	19	–
**Total**	**1029**	**735**	**4757**

408 (39.7%) of all primary operons could be experimentally validated, as a TSS could be assigned to the first gene of the corresponding operon. By analyzing the internal TSS, 735 sub-operons could be determined inside the 1029 primary operons. The majority (604) of the sub-operons consists of a single gene (Table 1, Supplementary Figure 3).

The largest primary operon contains 16 genes, which encodes mainly genes with no annotated function (“hypothetical protein”) [15] (Supplementary Table 1).

The number of monocistronically transcribed genes was determined to be 4757 (56.6% of all CDS), of which 1789 genes (37.6%) were associated with a TSS (Table 1).

#### Global identification of transcription start sites (TSS), 5′-UTR lengths and promoter consensus motifs in the *Actinoplanes* sp. SE50/110 genome sequence

For the analysis of growth-dependent transcription, a fundamental knowledge about the transcriptional landscape of the *Actinoplanes* sp. SE50/110 genome is required. The identification of transcription start sites (TSS) and corresponding promoters, which are only active in specific growth phases is useful for understanding regulatory processes and networks in *Actinoplanes* sp. SE50/110.

Based on the 5′ enriched library data from 15 and the whole transcriptome profile from this study, the positions of TSS was determined using the software ReadXplorer [30]. The automated prediction revealed 7937 TSS. Filtering and manual curation resulted in 4228 primary TSS, which could be assigned to 2787 CDS (33.2% of all annotated features) (Supplementary Table 2). This is a 3-fold increase to previous studies (1427 TSS assigned to 799 CDS) [35].

The 5′-untranslated region (5′-UTR) was determined as distance of TSS to the corresponding translation start site (TLS). Transcripts with a distance ≤3 nt were classified as leaderless transcripts. This results in 1179 TSS (14.03% of all CDS), which belong to leaderless transcripts, whereas 3049 TSS were assigned to transcripts carrying a 5′-UTR. The 5′-UTR length ranges between 4 and 494 nt, but 90% of all 5′-UTRs are less than 200 nt in length (Supplementary Figure 4).

Upstream of the identified TSS, promoter motifs could be found, such as the − 10 region (Pribnow box) and the − 35 region. Therefore, 50 bp upstream of each identified primary TSS were searched with the tool Improbizer [31]. For the − 10 region a conserved hexamer motif represented by TAnnnT was found in 4143 (98%) of all sequences examined (Supplementary Table 1). This result is in line with the findings from [35], analyzing the upstream sequences of 318 TSS in *Actinoplanes* sp. SE50/110.

In this study, the T on the first position of the identified hexamer was found in 63.6% of the analyzed sequences. For the A on second position within the − 10 motif, a frequency of 90.8% was determined. In the last position of the − 10 hexamer a T is present in 85.7% in the considered sequences in *Actinoplanes* sp. SE50/110. Therefore, the identified − 10 region perfectly matches the most highly conserved bases of the − 10 motif in the model organisms *Escherichia coli* [32] and *Streptomyces coelicolor* A3(2) [28]. The slightly overrepresented G at position − 13 indicates that some promoters feature an extended − 10 region [33].

**Fig. 3:**
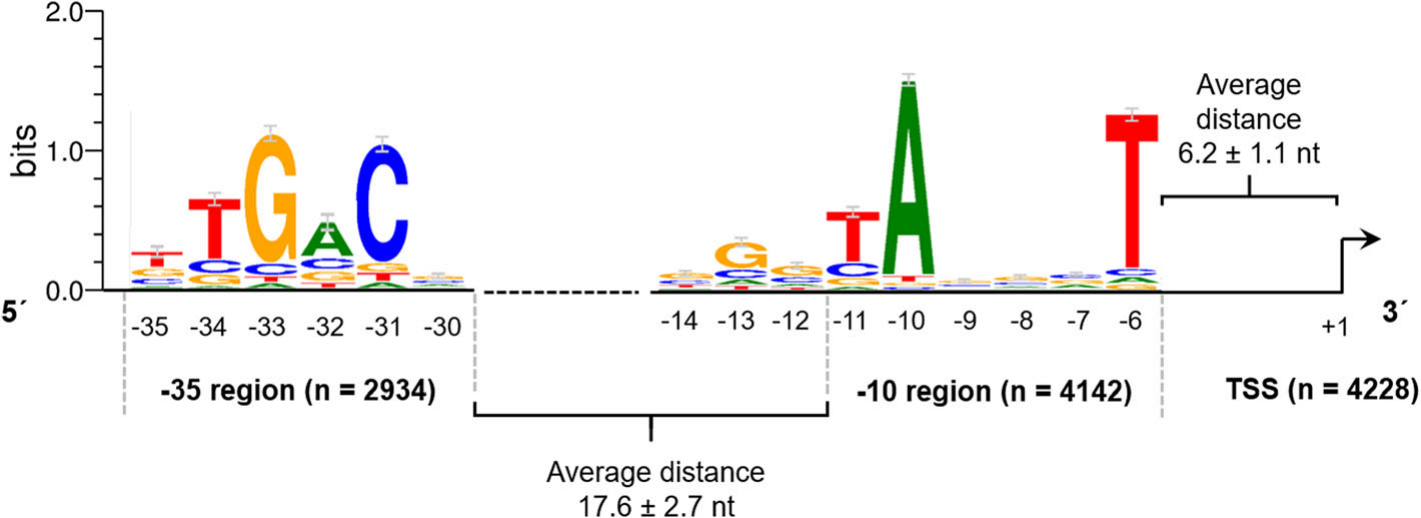
Conserved − 10 and − 35 regions identified in the promoter regions of the *Actinoplanes* sp. SE50/110 genome. The motifs were identified using Improbizer [31] searching upstream of 4228 TSS, resulting in 4142 putative − 10 regions and 2934 putative − 35 regions. The sequence logos were created using the software WebLogo (version 3.7.4) [34]

The average distance of the − 10 hexamer to the corresponding TSS was found to be 6.2 ± 1.1 nt, whereas 82% of all spacer lengths range between 5 and 7 nt (Fig. 3). The TSS itself is a purine in 75.4% of the cases (24.2% A and 51.2% G).

In the − 35 region the consensus hexamer nTGACn was determined in 2934 of all 4228 TSS upstream sequences (69.4%) using the software Improbizer [31], whereas the highest frequencies for G at position three (83.7%) and C at position five (82.0%) were found. However, the T at position two (67.9%) and the A at position four (53.0%) are less conserved in the *Actinoplanes* sp. SE50/110 –35 promoter region. This motif resembles the − 35 consensus motif of *E. coli*, which is TTGACA [32]. The average distance between the − 10 and the − 35 region was found to be 17.6 ± 2.5 nt for 2906 promoters, which contain both a − 10 and − 35 region. This spacer length is in common with the average distance of 17 nt described as optimal in *E. coli* consensus promoters [32].

In general, the promoter analysis is in accordance to the results described in the literature [15, 35]. However, in this study a much higher amount of data was used to determine the consensus motifs. Especially the consensus sequence regarding the − 35 region could be improved, as it is more related to the motifs described in the literature, e.g. for *E. coli* [32] or *S. coelicolor* A3(2) [28]. In addition to promoter analysis, for 93.2% of all analyzed leadered transcripts with a 5′-UTR-length > 10 nt a conserved ribosome binding site (RBS, Shine-Dalgarno sequence) could be found. The detected consensus motif is nGGAGn (Supplementary Figure 5).

#### Identification of co-transcribed genes by hierarchical cluster analysis of transcription dynamics data

**Fig. 4:**
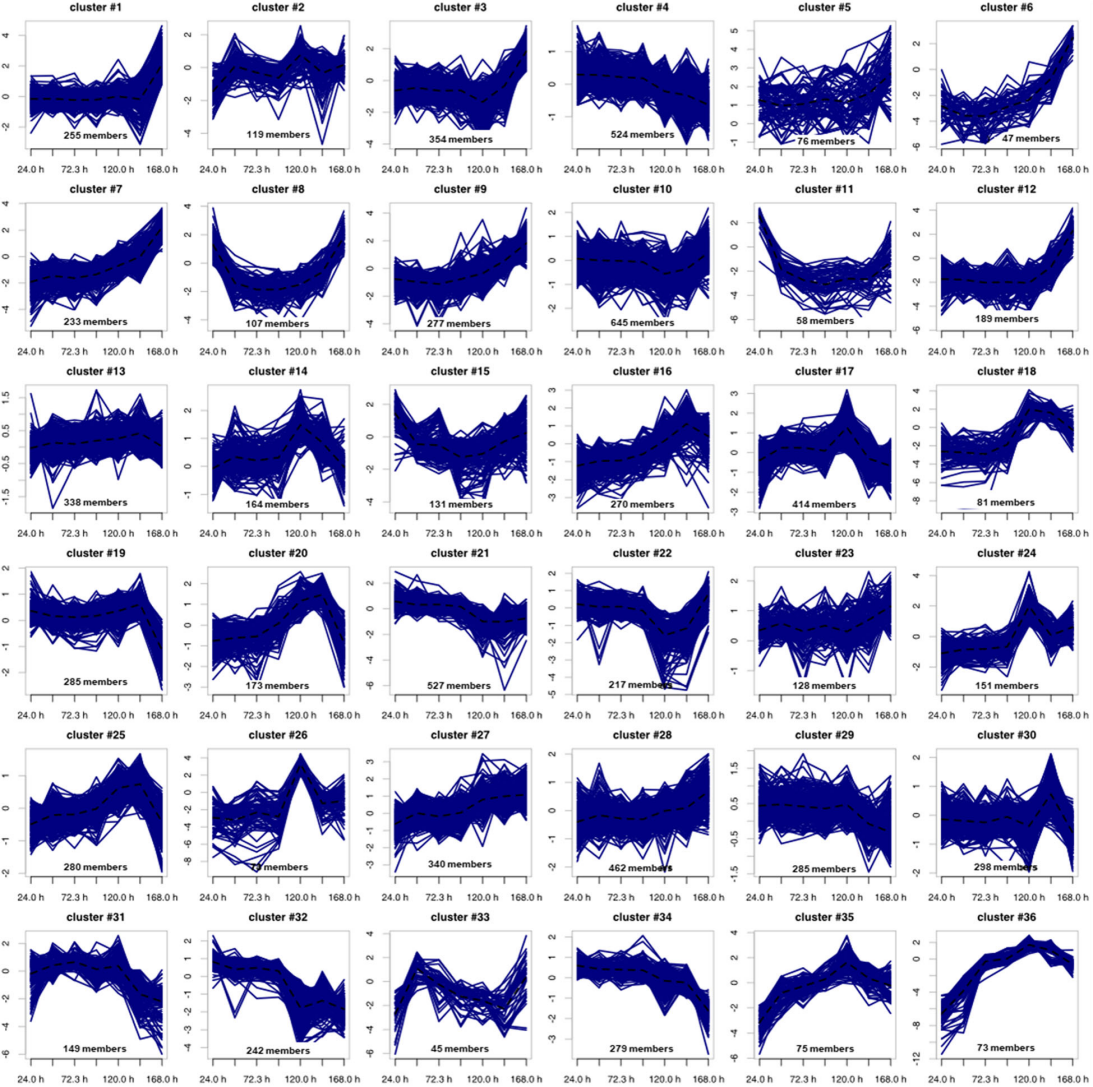
Hierarchical cluster analysis cluster analysis of transcription dynamics with calculation of optimal cluster size and cluster grouping using the tool Omics Fusion [36]. The log2(fold-changes) values for each time point and gene were used as input and are shown in the cluster graphs. The respective mean value of each cluster is visualized by a dashed line. For better visualization the trend of each cluster is scaled differently

In order to identify co-regulated genes, a hierarchical cluster analysis was performed using transcriptome data determined for each time point (Supplementary Table 3). The hierarchical cluster analysis was performed within the software Omics Fusion [36] and resulted in an optimal cluster amount of 36 (Fig. 4; Supplementary Figures 6 and 7). The clusters contain 45 to 645 genes.

An overview about predominant functions and pathways in a group of genes can be achieved by classification according to COG (clusters of orthologous groups) database [37] and KEGG (Kyoto Encyclopedia of Genes and Genomes) database [38]. Therefore, the cluster obtained from hierarchical cluster analysis can be examined for enriched gene functions and overrepresented pathways (Supplementary Table 3). Furthermore, strong changes in the course of transcription could be a hint for metabolic switches or different stages of the *Actinoplanes* sp. SE50/110 life cycle [39].

The earliest transcriptional change can be observed for genes, which are grouped in clusters 8, 11 and 15. These genes are highly transcribed in the lag phase, but almost no longer present during filamentous growth. Additionally, an increasing transcription in the late stationary phase could be observed for several genes of these clusters. Functional analysis of these three clusters revealed an enrichment of genes encoding chemotaxis, motility and flagellum associated proteins (COG class N; cell motility). More than 65% of the genes encoding proteins of these COG class in the *Actinoplanes* sp. SE50/110 genome are grouped in these three clusters. Since the cultivation was inoculated with spores generated on SFM agar plates, cell motility proteins are necessary and therefore highly transcribed in this stage of life cycle [39, 40]. Genes involved in flagellar development have been identified and described to be highly expressed in spores in *Actinoplanes* spp. [41, 42]. Due to the fact, that these genes are only expressed for a short time after inoculation, spore formation and cell motility seem to play a minor role in the further course of cultivation under the tested conditions. Interestingly, transcription of many of these genes increases in the late stationary phase. Therefore, it can be assumed that spores are formed at the end of the cultivation. So far, sporulation in liquid media could not be shown for *Actinoplanes* sp. SE50/110, yet. However, it was described for *Bacillus subtilis* [43] and *Streptomyces* spp. [44].

The clusters 4, 21, 32 and 34 with continuously decreasing transcript abundance are dominated by genes encoding ribosomal proteins and other proteins involved in protein biosynthesis (COG class J; translation, ribosomal structure and biogenesis). More than 60% of these features are located in these clusters. Considering only the 30S and 50S ribosomal proteins, these are almost only distributed among clusters 32 and 34. The corresponding profiles closely match the pattern of the stringent response in other close related actinobacteria, like *S. coelicolor* A3(2) [45] or *Corynebacterium glutamicum* [46, 47]. A continuously decreasing transcript level of ribosomal proteins and other proteins with functions related to the protein biosynthesis fits well to the expectations of an enhanced translation machinery for boosting cell growth at the beginning of the growth phase. The transcription of these genes decreases during cultivation reaching the lowest level in the stationary phase at which growth stops. This effect was previously described in *S. coelicolor* A3(2) [29].

Typical for actinomycetes is an increased production of secondary metabolites in the stationary phase, which is reflected by the transcription of the respective genes [28, 29]. The genome of *Actinoplanes* sp. SE50/110 harbors 20 predicted biosynthetic gene clusters for secondary metabolites, including the acarbose biosynthetic gene cluster [15]. Genes which are associated with one of these gene clusters were identified with antiSMASH 5.0 [48]. Most of these predicted secondary metabolite gene clusters are highly transcribed in the transition phase (clusters 17, 20 and 30; Fig. 4) and in the stationary phase (cluster 1, 3, 7, 10, 12 and 16; Fig. 4). Therefore, for 14 of all 20 predicted secondary metabolite gene clusters, an increased transcription during the late growth phase and stationary phase could be shown. They encode for terpene (carotenoid), NRPS, PKS, lassopeptide, lantipeptide, bacteriocin, or melanin biosynthesis. These findings indicate a typical switch from primary to secondary metabolism described for most organisms [49, 50]. In *S. coelicolor* A3(2) similar effects were observed by analyzing the transcription of secondary metabolite gene clusters in a growth-dependent manner [29].

The six remaining secondary metabolite gene cluster encode two siderophore, a terpene, a pyochelin and the acarbose biosynthesis and display different transcription dynamics. The two siderophore biosynthesis gene cluster differ from each other regarding their transcriptional course: For the first one, an increased transcription both during lag and stationary phase (cluster 15; Fig. 4) was observed, whereas the second one shows a slight increase of transcription during the stationary phase (cluster 3; Fig. 4). This could indicate different needs for iron in the growth versus stationary phase. In contrast to the beforementioned carotenoid biosynthesis gene cluster, the second terpene cluster was found to be highly transcribed during lag and stationary growth phase showing a similar transcription course as several cell motility and spore formation genes (cluster 8; Fig. 4). However, further investigation has to be made regarding the metabolic product of this gene cluster to determine a potential connection to e.g. sporulation.

The pyochelin biosynthesis gene cluster was found to be transcribed similar to the growth curve (cluster 36; Fig. 4). An increasing transcription was observed during growth, but no further increase could be found during transition and stationary phase. The gene products of pyochelin biosynthesis were analyzed in previous studies regarding their sub-cellular localization. The results revealed a close connection to the bacterial cell membrane [51]. The same localization was identified for the products of the *acb* gene cluster. However, transcription of the *acb* genes was found to be increased in the early growth phase and decreases until cells reaching the stationary phase (clusters 31 and 32; Fig. 4).

Due to the fact, that the transcription is increased in the early growth phase, the *acb* gene products seem to be more important for cell metabolism and therefore do not qualify as genes involved in secondary metabolite biosynthesis. The expression dynamics of the *acb* genes over the course of the cultivation will be discussed in detail in the following.

### Analysis of proteome data during the whole cultivation process

#### Processing and filtering of proteome data

To investigate the expression dynamics of *Actinoplanes* sp. SE50/110 proteome analysis was performed at all seven time points. Proteins were isolated from *Actinoplanes* cells (cytosolic fraction) and from the supernatant (extracellular fraction) and proteins were measured using state-of-the-art mass spectrometry (QExactive mass spectrometer). This resulted in a total number of 2675 proteins (32.3% of all annotated CDS), whereas 2496 were identified in the cellular fraction and 878 were found in the extracellular fraction. Principal component analyses (PCA) were performed to check the quality of the proteome data (Supplementary Figure 8 and 9).

Out of 878 proteins identified in the extracellular fraction 699 (79.6%) could also be detected in the cellular fraction. According to previous protein localization predictions [51] of these 699 proteins identified in both fractions 534 could be assigned as cytosolic, 103 are membrane associated or located at the inner membrane and 53 are previously predicted as extracellular proteins since a signal peptide could be identified in the amino acid sequence of the respective protein. Furthermore, 36 of the 179 proteins exclusively identified in the extracellular fraction were predicted as cytosolic, 64 were predicted as membrane associated or inner membrane proteins and 71 proteins were predicted as extracellular proteins [51]. The identification of proteins predicted as cytosolic in the extracellular fraction was reported previously for several bacteria [52, 53]. Additionally, it could be shown for *Bacillus subtilis* that only 21% of over 900 extracellular identified proteins show a signal peptide [53]. In this study, for 124 (14.1%) of the 878 proteins identified in the extracellular fraction, a signal peptide was predicted, which is in good accordance to the literature. Proteins which were identified in both extracellular and cytosolic fraction as well as predicted as extracellular proteins [51], were excluded from the cellular fraction data set and kept in the extracellular data. These proteins were assumed to be genuine extracellular proteins due to their predicted signal peptide.

The different filter steps resulted in 2234 proteins, of which 1654 were identified in the cellular, 183 in the extracellular and 397 proteins were found in both fractions. In this way, for the cellular fraction for 1468 proteins (71.6%) data could be obtained for at least 6 of 7 time points. In the extracellular fraction for 240 proteins (41.4%) data are available for at least 6 time points (Supplementary Table 4). Differential expression analysis was performed according to processing of transcriptomic data. A schematic overview on processing and filtering steps can be found in Supplementary Figure 10.

#### Overview of proteome dynamics in *Actinoplanes* sp. SE50/110

**Fig. 5:**
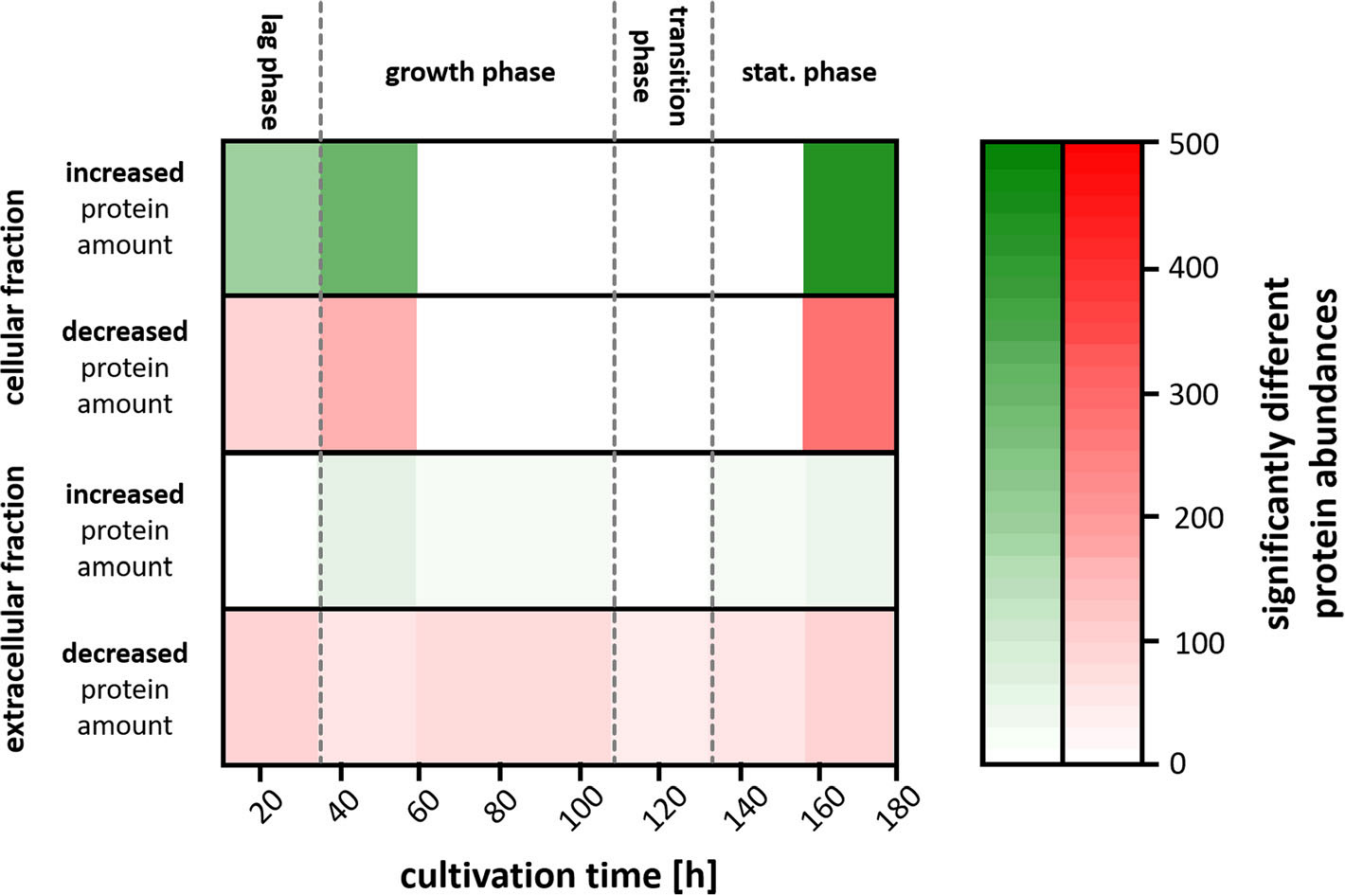
Overview about proteome dynamics in *Actinoplanes* sp. SE50/110. Number of proteins with significantly (p_adj_-value < 0.05) increased (green) and decreased (red) protein abundances during cultivation at the given time points in the cellular and extracellular fraction. Growth phases are indicated with dashed lines

The whole proteome analysis revealed 2234 different proteins, which could be detected in the cellular and extracellular fraction of *Actinoplanes* sp. SE50/110. Applying differential expression analysis 1441 proteins (1374 cytosolic and 67 extracellular) could be identified, which show a significant difference (p_adj_ < 0.05) for at least one time point. The number of significant different protein amounts compared to the respective mean value (protein fold-change) changes during the cultivation process. Figure 5 shows the total amount of genes with a significant difference regarding the respective protein amount.

Strikingly, the extracellular proteome fraction shows only a small number of significant different protein levels. This shows higher stability of extracellular proteins compared to cytosolic proteins due to absence of proteases or other influences [54].

For the cellular fraction clearly changes of the proteome repertoire during growth could be observed. The highest number of significant different protein amounts was observed in the late stationary phase after 168 h with 428 increased (29.7%) and 278 decreased (19.3%) proteins. Furthermore, a high number of significantly different protein abundancies was detected at the beginning of the cultivation in the lag phase (190 increased and 89 decreased proteins) and in the early growth phase (292 increased and 150 decreased protein amounts). These findings are in common with the overview on transcript level (Fig. 2), in which highest numbers were also observed in the lag and stationary phase.

Interestingly, nearly no significant differences on proteome level were observed during filamentous growth and transition phase. This shows a stable protein repertoire of the cells during filamentous growth, which is in common with the transcription profile (Fig. 2). Both on transcriptional and proteome level strongest changes could be observed in the lag phase and in the stationary phase, where secondary metabolism could be observed. A slight offset between transcriptional and proteomic changes can be explained by the time of translation, since changed transcript level is necessary before changes of protein level can occur [55]. Minimal number of significantly different protein abundancies was observed between 72.3 h and 144 h. In accordance to that, the minimal number of differentially transcribed genes was found in the time between 47.3 h and 96 h, which reflects the offset of one time point (24 h) between transcription and translation.

#### Identification of different post-translational modifications by comprehensive proteome analysis

Post-translational modification is an important mechanism for regulation of protein activity, localization or stability. To get an overview on different protein modifications in *Actinoplanes* sp. SE50/110, peptide sequences were analyzed for all known modification types using MaxQuant software [56]. This resulted in the following modification types: Oxidation, acetylation, phosphorylation and glutamine (Gln) converted into pyroglutamic acid (pyro-Glu). All other modifications were low abundant or not significant. It has to be noted, that phosphorylation sites could not be determined in detail since phospho-proteome analysis requires specific sample preparation. However, 55 proteins were found to be phosphorylated at serine, threonine or tyrosine residues.

The whole proteome analysis of *Actinoplanes* sp. SE50/110 revealed a number of 821 (30.7% of all detected proteins) proteins which are post-translationally modified at different positions. This finding matches the results for other bacteria obtained from the literature, e.g. *Leptospirillum* spp. [57]. However, under stress or nutrient-limiting conditions, bacterial proteomes were found to be more often modified on a post-translational level [58, 59].

In total, 176 proteins were found to be acetylated at their respective N-terminus. 380 proteins containing oxidations at methionine residues. This modification was identified up to three times per peptide. However, most of the modifications occur only once per protein. Finally, conversion of glutamine to pyroglutamic acid was observed in 415 proteins. All post-translational modifications and their respective positions can be found in Supplementary Table 4.

Interestingly, several proteins encoded by the *acb* gene cluster were shown to be modified. An overview on Acb protein modifications is given in Table 2.

**Table 2:** Overview on post-translational modifications (without oxidations) of Acb proteins of *Actinoplanes* sp. SE50/110 during growth

Protein	Annotated function	Modification(s)	Time Point(s)
AcbZ	acarbose-resistant alpha-amylase	Gln → pyro-Glu	T1; T3; T4; T6
AcbW	ABC-type transporter; ATPase	Gln → pyro-Glu	T4; T6
AcbU	1-epi-valienol-7-phosphate 1-kinase	Gln → pyro-Glu	T3
AcbR	1-epi-valienol-1,7-bisphosphate-1-adenylyltransferase	Gln → pyro-Glu	T4
AcbQ	acarbose 4-alpha-glucanotransferase; amlyomaltase	Acetylation Gln → pyro-Glu	T3 T5
AcbO	2-epi-5-epi-valiolone-7-phosphate 2-epimerase	Gln → pyro-Glu	T3
AcbE	acarbose-resistant alpha-amylase	Gln → pyro-Glu	T3; T4
AcbD	acarviose transferase	Gln → pyro-Glu	T2; T5; T7

Interestingly, the only N-acetylation among Acb proteins was identified in the putative acarbose 4-alpha-glucanotransferase AcbQ during the middle of the growth phase (T3; 72.3 h). Since N-terminal acetylation can affect the protein stability in both directions [60–64], it can be assumed that stability of AcbQ is post-translational influenced. Interestingly, AcbQ shows one of the most stable protein abundances among Acb proteins over the cultivation process (Fig. 8). This could indicate, that AcbQ possibly plays an important role in the physiology of *Actinoplanes* sp. SE50/110, e.g. within the acarbose metabolism. However, the specific function of AcbQ in the acarbose biosynthesis pathway has not yet been proven [13, 18]. Nevertheless, if AcbQ is an important enzyme in *Actinoplanes* sp. SE50/110 preventing its degradation is a possible action to increase production by the cell [65, 66]. It is notably, that most of the glutamine to pyroglutamic acid modifications of the Acb proteins were identified during the filamentous growth phase (72.3 h and 96.5 h). This could be a hint for altered enzymatic activity during filamentous growth caused by this modification. Nevertheless, this has to be proven by further experiments.

### Combining transcriptome and proteome data to elucidate expression dynamics of *Actinoplanes* sp. SE50/110 using a combined clustering approach

The expression of genes in bacteria is regulated on transcriptional, post-transcriptional, translational and post-translational level. By combining transcriptome and proteome data of each gene, correlation of transcription and translation could be performed. However, if transcriptome and proteome data do not correlate in an expected manner different regulatory stages could be responsible for that, such as protein degradation [55].

Pearson correlation of each available transcript/protein data pair was obtained. The overall Pearson coefficient was found to range from 0.10 to 0.63. In previous studies Pearson correlation coefficients of about 0.4 to 0.5 were reported for correlation of transcriptome and proteome data in bacteria [55, 60, 61], and between 0.66 and 0.76 for *Saccharomyces cerevisiae* [62]. Compared to this, the transcript/protein data pairs of *Actinoplanes* sp. SE50/110 display a broad range of correlation. Weak correlations can be referred to technical and methodological constrains, but also to translational and post-translational regulation processes [63, 64]. Therefore, correlation of transcription and protein abundance is often poor [55, 67].

For the different growth phases the differences for the respective transcriptome and proteome data were obtained. In the lag phase (24.0 h) a correlation of 0.44 was observed. Strongest correlation between transcriptome and proteome data was found in the early growth phase (47.8 h and 72.3 h) with a Pearson correlation coefficient of 0.63 and 0.48 respectively. Lowest correlation was observed when the cells entered the early stationary phase (120.0 h) with a Pearson coefficient of 0.10. Interestingly, the difference between transcriptome and proteome data is less when comparing transcriptomic data from time point X to proteome data from time point X + 1 meaning to compare transcriptome data from 24.0 h with proteome data from 47.8 h, and so on. This results in Pearson correlation coefficients of 0.61, 0.61, 0.50, 0.24, 0.21 and 0.20 respectively. These findings indicate the offset between transcription and translation caused by protein folding and processing. Furthermore, proteins are more stable, and their half-life time is much longer compared to the corresponding mRNA [68, 69].

**Fig. 6:**
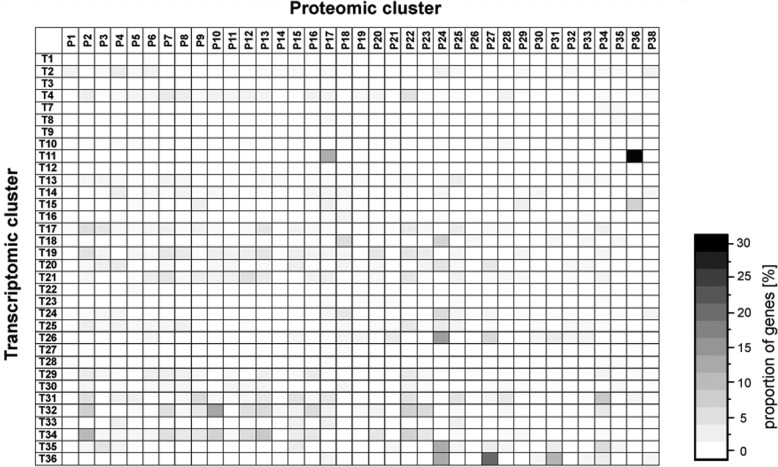
Connected heatmap of clustered transcriptome and proteome dynamics in *Actinoplanes* sp. SE50/110. Transcriptome clusters (T) are arranged vertically and proteome clusters (P) are arranged horizontally. Strong color indicates a high proportion of genes of the corresponding transcriptomic cluster which are present in the respective protein cluster

Transcriptome and proteome data were compared using a combined clustering approach via connected heatmaps. Therefore, only genes of which both transcriptome and proteome data are available were considered (2050 genes). The cluster analysis of the proteome data resulted in an optimal cluster amount of 37 proteome clusters (Supplementary Figures 11 and 12) connected to 34 of the 36 transcriptome clusters identified previously (Fig. 6). The transcriptomic cluster 5 and 6 are not included in this analysis, since no proteome data were obtained for genes inside this cluster, which contains of several hypothetical proteins, a few transcriptional regulator gene as well as tRNAs and rRNAs. Since tRNAs and rRNAs of course have not protein data and regulators are often low expressed, it is not surprising that no protein data are available for the genes in transcriptome cluster 5.

The resulting transcript and protein clusters were compared to each other regarding co-occurrences. This way, genes with both the same transcription pattern (co-transcribed) and the same protein dynamic can be identified. However, the respective transcription and protein trend can differ. The results are given in percentage of the respective transcriptome cluster size (Fig. 6).

It is striking, that several transcriptomic clusters are distributed over a lot of protein clusters (e.g. clusters T4, T17, T19, T21, T32 and T34), since only a few co-occurrences could be found. A reason for that could be the large cluster size and the functional diversity of the genes inside these clusters.

The highest similarity was observed for genes located in transcription cluster T11 and proteome cluster P36. In this cluster pair predominantly genes of sporulation, chemotaxis and motility can be found. The remaining genes of cluster 11 of which a protein could be detected are grouped in protein cluster P17. Both protein clusters show a strong protein signal in the lag phase (cluster P36) or early growth phase (cluster P17). This is in common with the transcription dynamics of these genes grouped in cluster T11, which show an increased transcription in the lag phase. This shows a close connection of transcript and protein abundance of genes involved in chemotaxis, sporulation, flagellar biosynthesis and motility (COG class N).

Further co-occurrences were observed in clusters T32 and P10 (Fig. 6). These clusters mainly consist of ribosomal proteins and other translation related genes and proteins (COG class J). Both clusters show a continuously decreasing transcript and protein abundance during the cultivation process. This shows, that changes on transcript level have an immediate effect on proteome level. Ribosomal proteins seem to be mainly regulated on transcriptomic level, since protein dynamics is highly similar to transcriptional changes. On transcriptional as well as on proteome level decreasing signals were observed for about 50% of all annotated ribosomal proteins in the *Actinoplanes* sp. SE50/110 genome. However, some of the co-transcribed ribosomal proteins could be found in other protein clusters, like P23, which show a more constant protein level during filamentous growth. This shows that some ribosomal proteins are more stable than others.

Interestingly, the proteins of the acarbose biosynthesis gene cluster are distributed over 5 different cluster (P5, P13, P15, P28 and P34), although the *acb* genes were found to be highly co-transcribed in transcriptomic clusters T31 and T32. This indicates a regulation of *acb* gene expression on a post-transcriptional level and at least different protein half-live times. These findings will be discussed in the next chapter more detailed.

### The genes of the acarbose biosynthetic gene cluster are transcriptionally and post-transcriptionally regulated during filamentous growth

**Fig. 7:**
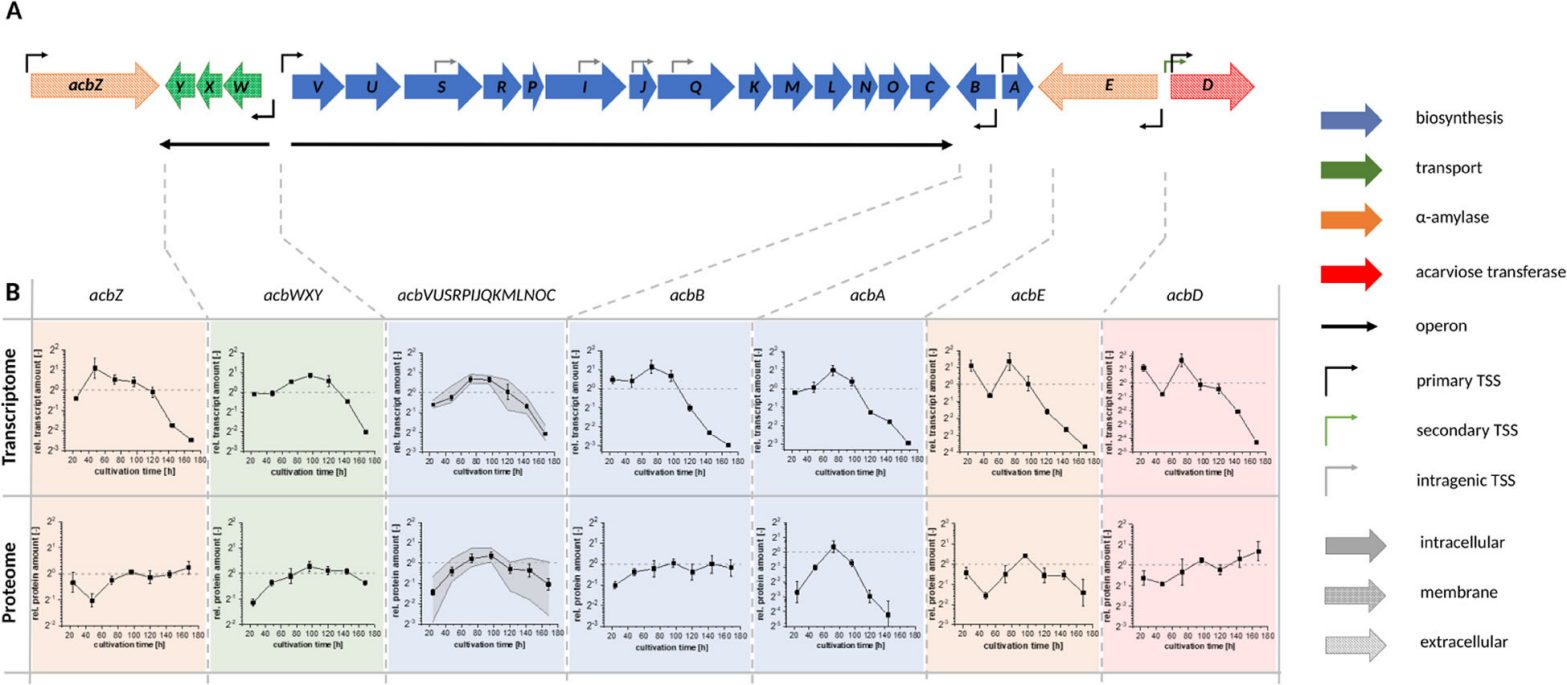
Overview about the expression dynamics of the *acb* gene cluster in *Actinoplanes* sp. SE50/110. (A) The *acb* gene cluster with its transcriptional landscape including operon structure and TSS [15]. The function of all genes and operons are color-coded. The sub-cellular localization (according to [51]) of the corresponding gene products are encoded by filled, dotted and striped arrow content. (B) Dynamics of the relative transcript abundances and the relative protein amounts of the acb genes and Acb proteins. A relative abundance of one corresponds to the average amount of RNA or protein over all time points. Mean values and standard deviation of three biological replicates are shown for each time point. For the operon *acbWXY* only protein abundancies of AcbW are shown. For the operon *acbVUSRPIJQKMLNOC* the maximum and minimum values are shown by grey area

When analyzing trends over the time course for the differentially transcribed genes, those of the acarbose biosynthetic gene cluster were particularly striking. As shown above, the temporal transcription dynamics of the *acb* genes (Fig. 7b) seem to be highly similar to the specific acarbose formation rate (Fig. 1b). Especially the genes *acbZ*, *acbB* and *acbA* follow the course of acarbose formation rate with an increase during the first 48 h and continuous decrease afterwards. This trend was observed for all further *acb* genes as well, but less strong.

The genes *acbZ, acbB, acbA, acbE* and *acbD*, which represent the monocistronically transcribed genes in the *acb* gene cluster, were grouped within a cluster of genes with substantial decrease of transcript amounts over the growth curve (Fig. 4, cluster 32). All other *acb* genes cluster with genes showing a slight increase until late growth phase followed by a decrease of transcript amounts (Fig. 4, cluster 31). Consequently, lower transcript levels were measured in the stationary phase compared to the filamentous growth phase for all *acb* genes (Fig. 7). Remarkably, the most distinct differences were detected for the genes coding for the extracellular proteins AcbE (acarbose-resistant alpha-amylase) and AcbD (acarviose transferase) with fold changes of 32.7 and 60.9 on transcriptomic level, when comparing the filamentous growth (72.3 h) and the stationary phase (168 h). The genes *acbB* (coding for dTDP-4-keto-6-deoxy-glucose dehydratase) and *acbA* (encoding dTDP-glucose synthase) show fold changes of 17.4 and 15.2 comparing the filamentous growth phase and the stationary phase.

For all other *acb* genes, fold changes between 4.9 and 9.7 were found on transcript level. These *acb* genes code for the proteins of acarbose biosynthesis, an exporter and the extracellular alpha-amylase (*acbZ*). The fold change of *acbZ* was determined as 8.0 comparing the growth and the stationary phase. However, it was grouped in cluster 32 together with *acbB*, *acbA*, *acbE* and *acbD*.

The similar course of transcription during growth and similar fold changes between growth and stationary phase indicate a co-regulation of these genes. Although the genes *acbZ*, *acbB*, *acbA*, *acbE* and *acbD* are transcribed monocistronically, co-regulation was reported for the pair *acbD* and *acbE* [25]. The genes *acbE* and *acbD* as well as *acbB* and *acbA* are located in opposite directions to each other in the genome sharing an intergenic region (Fig. 7). It was assumed that the intergenic regions between the pairs *acbA* & *acbB* and *acbD* & *acbE* harbor binding sites for transcriptional regulators, which explains the co-regulation of these genes [13, 70]. For *acbE* and *acbD* it was shown, that the MalR type transcriptional regulator AcrC (ACSP50_6387) is the repressor of these two genes in *Actinoplanes* sp. SE50/110 [25].

All other *acb* genes, encoding for intracellular acarbose metabolism and acarbose export, are transcribed in the two operons *acbVUSRPIJQKMLNOC* and *acbWXY* (2.2.3). These two operons, which are also located in a head-to-head arrangement, seem to be co-regulated as well. So far, no regulators of these two operons are described.

Noticeably, the course of the transcript abundance for all *acb* genes (Fig. 7) are more or less in accordance with the course of the specific product formation rate (Fig. 1b). This might be an indication that the transcription of these genes has a direct influence on the acarbose production. However, protein abundances are not in correlation with the specific product formation rate for all Acb proteins. Especially the alpha-amylase AcbZ, the dTDP-4-keto-6-deoxy-glucose dehydratase AcbB and the acarviose transferase AcbD are not following their respective transcript signals on protein level. Protein levels for these enzymes seem to be almost constant during cultivation process. This could be a hint for a post-transcriptional regulation or high protein stability due to the secretion of these proteins, since less protease activity is expected in the extracellular space.

Based on the literature [18] and current models [26] acarbose is formed intracellularly and the secreted proteins AcbZ, AcbE and AcbD are not essential for acarbose formation [26]. The acarbose-resistant alpha-amylases AcbE and AcbZ degrade starch and maltodextrins to maltose and maltotriose or higher malto-oligosaccharides in the extracellular space [71]. The gene *acbD* encodes an acarviose transferase, which is supposed to catalyze the transfer of acarviosyl moieties from acarviose metabolites to the hydroxyl group of various sugars [72, 73]. Therefore, a direct correlation of the expression of the genes *acbZ*, *acbE* and *acbD* with the acarbose formation was not expected. However, it could be shown in previous studies, that AcbD is essential for acarbose formation in *Actinoplanes* sp. SE50/110 since an Δ*acbD* deletion mutant shows no acarbose formation [74]. As AcbD is proposed to transfer sugar moieties onto acarbose it can be assumed, that AcbD expression is important during the whole cultivation process and therefore should be expressed constantly. However, protein abundance of AcbD was found to be even slightly increased during growth. In contrast, *acbE*, which is transcribed highly similar to *acbD*, shows a decreasing protein abundance in parallel to its transcription. This difference of protein abundances of the transcriptionally co-regulated genes *acbE* and *acbD* could be explained by the fact, that *acbD* seem to be transcribed from two or even three different TSS with different leader transcripts [15], which can influence AcbD translation efficiency (Fig. 7).

Interestingly, AcbB, which is involved in the synthesis of dTDP-4-amino-4,6-dideoxy-D-glucose, is the only intracellular acarbose biosynthesis enzyme whose protein dynamics highly differs from its transcription profile during growth. The transcription of *acbB* strongly decreases during cultivation whereas its protein abundance stays on a constant level. In contrast, *acbA,* which seem to be highly co-regulated with *acbB* on a transcript level, shows a different protein dynamic which seems to be coupled to the corresponding transcription signal. It is striking that the expression pattern of AcbB and AcbA differ, although they are involved in the same part of acarbose biosynthesis [19]. Differences in expression strength could not be explained by differences in ribosome binding sites, since both genes are transcribed leaderless [15]. Therefore, an almost constant protein level could be due to a higher protein stability of AcbB or a regulatory effect on protein level. Protein modifications were not found for neither AcbB nor AcbA.

Since AcbA shows strongest decrease on proteome level, it would be an interesting target for overexpression as low AcbA amounts might be a bottleneck in the pathway operated by AcbA, AcbB and AcbV.

**Fig. 8:**
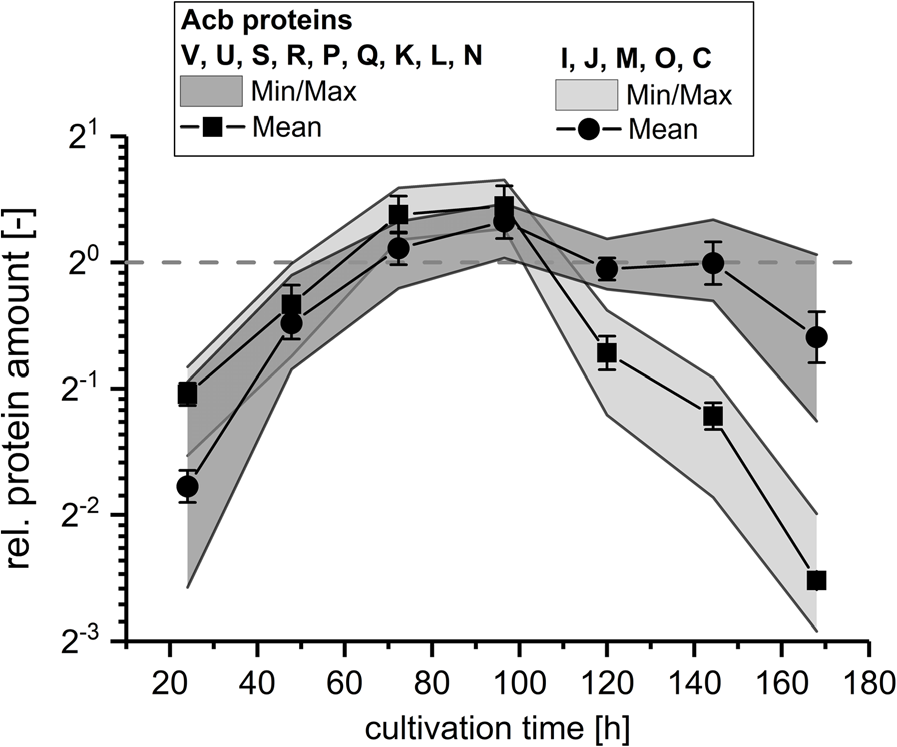
Dynamic of the protein abundancies of the Acb proteins encoded by the operon *acbVUSRPIJQKMLNOC*. Proteins were divided into two groups according to their behavior in the transition and stationary phase

Strikingly, the proteins encoded by the large operon *acbVUSRPIJQKMLNOC* show diverse abundancies on protein level until the transition phase (Fig. 8) at which acarbose formation decreases (Fig. 1a), whereas their respective transcription seem to be similar (Fig. 7).

The proteins AcbI, AcbJ, AcbM, AcbO and AcbC show a stronger decreasing protein abundance between 96 h and 168 h, whereas the other proteins encoded by the operon *acbVUSRPIJQKMLNOC* only slightly decrease. This could be due to different protein half-life times. Nevertheless, this could be an indication, that the five proteins AcbI, AcbJ, AcbM, AcbO and AcbC might be responsible for the decreasing acarbose formation during transition and stationary phase. Since AcbC, AcbO and AcbM catalyze the first steps in acarbose biosynthesis, down-regulation of the acarbose formation due to decreasing protein amounts might be beneficial to save energy and resources. This might also give explanation for the decrease of AcbA protein abundance during the transition and stationary phase, since AcbA catalyzes the first step of the second synthesis branch of the acarbose biosynthesis [19]. Additionally, this step is in competition to reactions of central metabolism in *Actinoplanes* sp. SE50/110, as D-glucose-1-phosphate serves as a substrate in other cellular processes. Furthermore, a homologous gene (*ACSP50_3024*) was identified in the genome of *Actinoplanes* sp. SE50/110 [75]. This gene was found to be transcribed constantly over the whole cultivation process with a slight increase in the stationary phase (cluster 10). Therefore, it can be assumed, that available D-glucose-1-phosphate is consumed by ACSP50_3024 and no substrate is available for further acarbose biosynthesis.

Due to this, it can be assumed that acarbose formation is blocked due to absence of the first steps of both branches of the acarbose biosynthesis pathway.

### Identification of genes co-expressed to the *acb* gene cluster

When analyzing genes co-transcribed to the *acb* genes located in one of the operons *acbWXY* and *acbVUSRPIJQKMLNOC* (cluster 31), it is noticeable that in addition to the *acb* genes only 120 other genes are located in cluster 31. Since most other clusters harbor more members, this indicates that the transcriptional dynamic of the *acb* genes is not rare but also not common in *Actinoplanes* sp. SE50/110. Analyzing the genes co-transcribed to these *acb* genes in cluster 31, also the genes *galG* and *galF* are among the genes with this transcription dynamics. The ABC-transporter GalHFG was formerly proposed as an acarbose importer, but it was shown that acarbose binds with low affinities to GalH. GalH has a high binding affinity to galactose, wherefore GalHFG is now suggested as a putative galactose importer [76]. The observation that these genes are co-regulated with the *acb* gene cluster and the direct genomic proximity might be an indication that these genes are after all involved in the acarbose metabolism. However, further experiments and proofs, like deletion mutants of these genes are needed to question the current assumptions about these genes.

Of the 120 co-transcribed genes, 30 genes are annotated as “hypothetical proteins” and 22 as “uncharacterized proteins”. Furthermore, two transcriptional regulators (*ACSP50_0424*, *ACSP50_8200*), two two-component regulator systems (*ACSP50_2300*, *ACSP50_5226*) and a sigma factor (*ACSP50_7877*) could be found inside of cluster 31 indicating a co-transcription to the acarbose biosynthesis operons. These regulators could be interesting targets for gene deletions analyzing the effect on acarbose biosynthesis.

A further example is the operon *ACSP50_6408* to *ACSP50_6411,* which was found to be transcribed in the same course as the two operons in *acb* gene cluster. This operon encodes enzymes involved in the histidine metabolism (formation of ergothioneine from L-histidine). Ergothioneine has been described to be synthesized in many actinomycetes, cyanobacteria, methylobacteria and some fungi. It is described to be resistant to autooxidation and therefore enable survive of microbes under oxidative stress [77].

Interestingly, the gene *ACSP50_2474* encoding a maltose degrading enzyme (AmlE), which was previously identified to be essential for maltose utilization in *Actinoplanes* sp. SE50/110 [78], was identified to be co-transcribed to the two *acb* operons. In the related species *S. glaucescens* GLA. O the *amlE*-homolog was even identified as part of the *gac* acarbose biosynthesis gene cluster, which suggests a co-evolution in this species [78]. This shows the close connection between maltose and acarbose metabolism, since maltose seem to be essential for production of acarbose in *Actinoplanes* sp. SE50/110.

Furthermore, the gene *cgt* (*ACSP50_5024*) was also found to be transcribed parallel to the *acb* operons. The function of the gene product is unclear. Several functional analyses were carried out on the extracellular protein Cgt, but no enzymatic activity could be determined [79]. However, the deletion of *cgt* lead to an increase of acarbose formation in *Actinoplanes* sp. SE50/110 [79]. This effect is supposed to be caused by the reduced metabolic burden, since Cgt was identified to be one of the highest abundant proteins in *Actinoplanes* sp. SE50/110 [80–82]. The similar expression patterns shown in this study, support the suggestion, that by deletion of genes co-expressed with the *acb* gene cluster, the acarbose formation might be improved. To further reduce the metabolic burden in order to improve the acarbose formation, this study suggests deletion of the 52 genes without functional annotation (see above).

In contrast to cluster 31, in cluster 32 there are 237 genes beside the monocistronically transcribed *acb* genes (*acbA*, *acbB, acbD, acbE* and *acbZ*) showing the same transcription pattern. However, 51 of these 237 genes are annotated as “hypothetical” or “uncharacterized proteins”. A high number of ribosomal proteins is located in this cluster. This indicates a close connection of these *acb* genes to the primary metabolism of *Actinoplanes* sp. SE50/110.

Furthermore, 9 transcriptional regulators (*ACSP50_1631*, *ACSP50_2235, ACSP50_4697*, *ACSP50_5005*, *ACSP50_6401*, *ACSP50_6463, ACSP50_8007, ACSP50_8120* and *ACSP50_8287*), a two-component regulator system (*ACSP50_3744*, *ACSP50_3745*) and 2 sigma factor genes (*ACSP50_0644*, *ACSP50_6006*) were determined to show the same transcription dynamics as the monocistronic *acb* genes located in cluster 32. Several genes located in cluster 32 are involved in amino acid transport and metabolism (31), nucleotide transport and metabolism (19) and carbohydrate transport and metabolism (19). This shows, that most of the genes co-transcribed with the monocistronically transcribed *acb* genes belong to the central metabolism of *Actinoplanes* sp. SE50/110.

Using the data from combined clustering approach 21 different genes were found to be clustered regarding both transcript and protein abundance with the *acb* genes (Fig. 6). Of these genes, 6 are annotated as “uncharacterized proteins”. Among the remaining 15 genes, a glycosyl transferase gene (ACSP50_7756) was identified. It needs to be elucidated in future, whether this enzyme is involved in the acarbose biosynthesis or by-component formation. In addition, one transcriptional regulator (*ACSP50_0424*) was found to show similar transcript as well as protein profile. This regulator gene seems to be widespread in the family Micromonosporaceae but no specific function was reported, yet. Therefore, it would be interesting to further analyze these transcriptional regulators regarding a potential effect on acarbose biosynthesis since they are highly co-expressed with several *acb* genes.

## Conclusions

The combination of robust and controlled cultivation conditions with state-of-the-art transcriptomics and proteomics in a high temporal resolution is well suited to answer a variety of biological questions. The close connection of acarbose biosynthesis and growth could be elucidated.

Using the transcriptomic data, comprehensive analyses of the transcriptional landscape of *Actinoplanes* sp. SE50/110 were performed. Using high-quality RNA-seq data of different growth phases more than 99% of all genomic features were covered in the analyses. This way, the operon structure with 1029 primary operons, 4228 transcription start sites and a consensus promoter sequences (− 10 motif: TAnnnT; − 35 motif: nTGACn) of the *Actinoplanes* sp. SE50/110 genome were obtained.

Through high-accurate proteome studies, 1441 proteins could be identified under the tested conditions, of which 1374 were found in the cellular fraction and 67 in the supernatant of *Actinoplanes* sp. SE50/110.

In this study, co-regulations of genes during different growth phases and in correlation to their respective protein dynamics were shown. Especially for acarbose biosynthesis genes striking results regarding transcription and protein dynamics could be achieved. It could be shown, that transcription of the acarbose biosynthesis gene cluster is in close correlation to the specific product formation rate regarding acarbose. However, on protein level several differences were found. Unlike to the other Acb proteins, AcbZ, AcbB and AcbD show a protein dynamic which differ from their respective transcription pattern suggesting that these proteins are more stable or post-transcriptional regulated. AcbB in particular seems to play an important role in acarbose biosynthesis since protein level was found to be constant during whole cultivation process. However, AcbA which catalyzes the step directly before AcbB seems to be a limiting factor in this branch of acarbose biosynthesis as protein level of AcbA strongly decreases since the middle of the filamentous growth phase (72.3 h).

Finally, genes could be identified, which beforehand were not in the focus of acarbose research in *Actinoplanes* sp. SE50/110. The combined clustering approach revealed several genes which are strictly co-expressed with the *acb* gene cluster. In this context, the transcriptional regulator genes *ACSP50_0424* was described as interesting target for further analyses regarding a potential effect on acarbose biosynthesis, because strong co-expression to *acb* genes was found.

This approach of analyzing the expression dynamics can be applied to other strains and organisms as well as experimental settings, like the spike-in of nutrients, stress factors or substances enhancing product formation. Thus, the temporal transcriptional response upon induced changes in environmental conditions could be elucidated with the here established methodology.

## Methods

### Strains, media, and cultivation conditions

The bacterial strain *Actinoplanes* sp. SE50/110 (ATCC 31044) was grown on soy flour medium (SFM; 20 g L^− 1^ soy flour, 20 g L^− 1^ mannitol, 20 g L^− 1^ agar, pH 8, tap water), in NBS complex medium and maltose-containing minimal medium as described elsewhere [51]. Bioreactor cultivations of *Actinoplanes* sp. SE50/110 was carried out in three biological replicates. NBS medium was inoculated from glycerol stocks prepared according to 81. After 2 days of cultivation 300 μL was plated on SFM agar plates and incubated for 5 days at 28 °C to generate spores. The spores were harvested by adding 2 mL ddH_2_O on the plates and carefully detaching them with a cotton swab. One plate resulted in 1 mL of spore suspension. Spores from 30 plates were pooled and used for the inoculation of each bioreactor cultivation. For the cultivation 3 L DASGIP® Bioblock (Eppendorf, Hamburg, Germany) bioreactors with a working volume of 1.6 L were used. Cells were grown in maltose minimal medium at 28 °C. The pH was set to 6.7 and automatically controlled by the addition of 10% H_3_PO_4_ or 2 N NaOH respectively. Dissolved oxygen level was set to 30% controlled by stirrer speed and oxygen partial pressure in the air supply in a two-step cascade.

The cultivations were examined by determination of the cell dry weight and quantification of acarbose as described previously [25]. For both transcriptome and proteome analysis, 1 mL of the cell suspension was centrifuged for 15 s at 16.000 *g* and immediately frozen in liquid nitrogen. Cell pellets were stored at − 80 °C until further processed for RNA or protein isolation. For the extraction of extracellular proteins 10 mL of the cell suspension was centrifuged for 2 min at 4000 *g*. The supernatant was frozen and lyophilized thereafter.

### Acarbose quantification using HPLC measurement

Acarbose was quantified from the supernatant of *Actinoplanes* sp. SE50/110 by high-performance liquid chromatography. Therefore, 1 mL of the culture supernatant was centrifuged (20,000 *g*, 2 min) to remove residual biomass and other particles. Next, 200 μL of the supernatant was mixed with 800 μL methanol by vortexing and centrifuged again (20,000 *g*, 2 min) to remove precipitate. Afterwards, the samples were transferred to HPLC vials and analyzed in an Agilent 1100 HPLC system (G1312A Binary Pump, G1329A ALS autosampler, G1315A diode-array detector (DAD)) using a Thermo Fisher Scientific (Waltham, MA, USA) Hypersil APS-2 column (125 × 4 mm, particle size: 3 μm) heated to 40 °C. As a mobile phase 32% phosphate buffer (0.62 g L^− 1^ KH_2_PO_4_ and 0.38 g L^− 1^ K_2_HPO_4_ x H_2_O) (solvent A) mixed with 68% acetonitrile (solvent B) was used with an isocratic flow of 1 mL min^− 1^. 40 μL of each sample was injected and separated for 10 min. Acarbose was detected at 210 nm (reference of 360 nm) with a DAD detector and quantified from the peak areas with a calibration curve.

### Isolation of total RNA and RNA-seq

RNA was isolated with a Macherey-Nagel NucleoSpin® RNA Plus Kit (MACHEREY-NAGEL, Düren, Germany) in combination with an rDNase Set (MACHEREY-NAGEL, Düren, Germany) as described previously [21]. In brief, frozen cell pellets were resuspended in 500 μL LB-buffer and transferred to 2 mL lysing matrix tubes. Cell disruption was carried out in a homogenizer three times for 20 s at 6.5 m s^− 1^ and cooled on ice between steps. Subsequently, the cell suspension was centrifuged, and the RNA was extracted from the supernatant using the NucleoSpin® RNA Plus Kit in combination with rDNase Set for an on-column DNA digestion. After the clean-up the DNA-digestion was repeated in-solution. Residual DNA was tested negatively with two primer pairs binding to genomic DNA of *Actinoplanes* sp. SE50/110. Quality and quantity of the RNA were analyzed with an Xpose® spectrophotometer (Unchained Labs, Pleasanton, CA, USA) and an Agilent RNA 6000 Pico kit run on an Agilent Bioanalyzer 2100 (Agilent Technologies, Santa Clara, CA, USA).

For each time point the RNA of three fermenters was isolated and used for cDNA library preparation. The preparation of cDNA libraries were performed according to 15. Additionally, a pool was generated for each fermenter of all time points using equimolar amounts of RNA.

All cDNA libraries were sequenced using TruSeq kits (Illumina, San Diego, CA, USA) on an HiSeq1500 sequencer system (Illumina, San Diego, CA, USA) using a paired-end mode with 2 × 70 nt read length.

RNA-seq yielded about 1.39 to 4.96 million read pairs for the 21 libraries (t_1_ to t_7_ in 3 biological replicates) and 1.91 to 2.79 million read pairs for the library of the pooled RNA samples over all time points. The reads were mapped to the reference sequence (GenBank: LT827010.1) using bowtie2 v2.3.2 in the paired-end mode [83], resulting in 90.3 to 98.9% mapped reads. The coverage of each annotated feature (protein-coding genes and RNA genes) was determined using featureCounts [84]. Afterwards, the software ReadXplorer was used for visualization of the transcriptomic data [30, 85]. After coverage analysis, differential expression analysis was carried out using the tool DESeq2 [86], to compare the differences in transcription for each gene at each time point. As a reference a pooled RNA sample from all time points was used as a mean value of transcription over the whole cultivation process. Thereby, log_2_ (fold-changes) (M-values) for each transcript compared to the mean transcript amount for all time points was determined in three biological replicates. RNA-seq data have been deposited in the ArrayExpress database at EMBL-EBI [87] under accession number E-MTAB-8857.

### Extraction of proteins from *Actinoplanes* sp. SE50/110 cell pellets

For protein isolation, the freeze-dried cell pellet was dissolved in 500 μl of 100 mM ammonium bicarbonate (Honeywell Fluka) and transferred to 2 mL lysing matrix tubes (0.1 mm spherical silica beads, MP Biomedicals, Santa Ana, California, USA). The cell suspension was disrupted in a homogenizer (FastPrep FP120, Thermo Fisher Scientific, Waltham, MA, USA) three times for 20 s at 6.5 m s^− 1^ and cooled on ice between steps.

Next, the organic solvent trifluoroethanol (TFE) was used for isolation of the cytosolic proteins [88]. Therefore, the supernatant of the ribolysed cell pellets was transferred in low protein binding collection tubes (Thermo Fisher Scientific, MA, USA). 1% RapiGest (Waters Corporation, Milford, MA, USA), 100 μl of TFE (Honeywell Fluka, Morristown, NJ, USA) and 5 mM Tris(2-carboxyethyl) phosphine hydrochloride (TCEP) was added to the cell suspension. The sample was incubated for 60 min at 60 °C and inverted several times during that time. In a second incubation step 20 μL of 200 mM Chloroacetamid (CAA) was added and left for 90 min in the dark. Afterwards, 5 μL of 200 mM TCEP was mixed to the solution. The sample was inverted and incubated at room temperature for additional 60 min. In the next step, the proteins within the cell suspension were digested.

### Extraction of proteins from the supernatant of *Actinoplanes* sp. SE50/110

For extraction of extracellular proteins, the method developed by [80] was used. In brief, the proteins of the freeze-dried supernatant were isolated by phenol extraction with subsequent methanol precipitation and several washing steps with 70% ethanol.

After drying the extracellular proteins were resuspended in 200 μl TE-Buffer and 5 mM dithiothreitol (DTT) and incubated for 60 min at 60 °C. 20 μL of 200 mM iodacetamid (IAA) was added and left for 90 min in the dark. Next, 5 μL of 200 mM DTT was mixed to the solution and incubated for additional 60 min. The isolated proteins of the extracellular space were digested afterwards according to the proteins of the cellular fraction.

### Protein digestion and mass spectrometry measurements and data analysis

For protein digestion, the solutions were diluted with 435 μL of 100 mM ammonium bicarbonate and 435 μL of bidistilled water. In the next step, 5 μL Trypsin Gold (1 μg μL^− 1^) (Mass Spectrometry Grade, Promega, WI, USA) was added and the solution was incubated at 37 °C overnight. The digest was purified on the next day with Sep Pak C18 cartridges (Waters Corporation, Milford, MA, USA). The Sep Pak C18 cartridges was rinsed with 1 mL of solution B (65% Acetonitril, 35% bidistilled water; 0.1% TFA). The column was equilibrated with 1 mL of solution A (98% Acetonitril, 2% bidistilled water; 0.1% TFA). Next the digest mixed with 1 mL of solution A was added and run through the column slowly. Subsequently, the cartridges were washed with 1 mL of solution A. The proteome was eluted with 100 μL solution B in low protein binding collection tubes and dried in a vacufuge concentrator (Eppendorf, Hamburg, Germany). Next the dried peptide mixture was resuspended in 15 μL of solution A and measured with a Nano-Drop™ 2000/2000c (Thermo Fisher, MA, USA).

LC-MS/MS measurement were carried out using a QExactive mass spectrometer (Thermo Fisher Scientific, Waltham, MA, USA) online coupled to the LC system. The peptides were separated on a 25 cm steel column Acclaim™ PepMap™ 100 C18-LC-column with a particle size of 2 μm and a diameter of 75 μm (Thermo Fisher, MA, USA).

Identification and label-free quantification (LFQ) analysis was performed using the software MaxQuant with default settings and a false discovery rate of p_adj_ < 0.05 [56]. For identification, ms spectra were searched with Andromeda against the target-decoy protein sequence database of *Actinoplanes* sp. data from 15. Only unique peptides were used for the quantification. An oxidation of methionine (15.99 Da) were allowed up to three times per peptide. As static modification a carbamidomethylation of cysteine (57.02 Da) and the dynamic modification of the N terminus with an acetylation (42.01 Da) was allowed.

The statistical analysis of LFQ data obtained from MaxQuant was performed with Perseus 1.6.10.43 [89]. In total, 565,792 MS/MS spectra were recorded for the cytosolic fraction resulting in 210,637 identified peptide sequences corresponding to 2663 proteins. For the extracellular fraction, 243,903 MS/MS spectra were recorded resulting in 35,000 identified peptide sequences, which could be associated to 911 proteins. As a first filtering step, proteins which were identified with less than two unique peptides were excluded from further analyses. Next, all time points of a protein were ruled out, of which the respective protein could not be identified in all three replicates. LFQ intensities were normalized by z-normalization and afterwards log_2_ (fold-change) was calculated by comparing each time point against the mean protein intensity over all analyzed time points. Finally, a two-sample *t*-test was performed to identify significant differences in protein level for each time point for the respective protein. The false discovery rate was set to p_adj_ < 0.05.

The mass spectrometry proteomics data have been deposited to the ProteomeXchange Consortium [90] via the PRIDE partner repository [91] with the dataset identifier PXD017973.

### Hierarchical cluster analysis

For hierarchical cluster analysis, the multi-omics data integration tool set *Omics Fusion* (https://fusion.cebitec.uni-bielefeld.de/) was used [36]. Genes were considered for analysis, when at least two third of the time points had numerical values. Missing values for single time points were replaced by means of the earlier and later time point. Hierarchical cluster analysis with grouping of clusters were performed with the Wards method for linkage and Euclidean distances, respectively. The maximum number of clusters was set to 50 and the optimal cluster size was calculated using the Krzanowski-Lai index [92, 93].

First, both transcriptomic and proteomic data sets were clustered individually. In the second step a combined clustering approach was used. Therefore, only features of which both transcriptomic and proteomic data are available were used. Again, Wards linkage method with Euclidean distance was used. The maximal cluster number was set to 50. As a result, a combined heatmap was generated indicating co-occurrences of features in the respective proteomic and transcriptomic cluster.

## Supplementary Information

**Additional file 1 Supplementary Figures**. Includes all supplementary figures.

**Additional file 2 Supplementary Table 1.** Operon structure of the *Actinoplanes* sp. SE50/110 genome.

**Additional file 3 Supplementary Table 2.** Transcription start sites (TSS) and promoter motifs in the *Actinoplanes* sp. SE50/110 genome.

**Additional file 4 Supplementary Table 3.** Transcription dynamics of *Actinoplanes* sp. SE50/110.

**Additional file 5 Supplementary Table 4.** Proteome dynamics of *Actinoplanes* sp. SE50/110.

## Data Availability

The transcriptome datasets generated for this study can be found in the Array Express database (www.ebi.ac.uk/arrayexpress) under accession number E-MTAB-8857. The mass spectrometry proteomics data have been deposited to the ProteomeXchange Consortium (http://proteomecentral.proteomexchange.org) via the PRIDE partner repository with the dataset identifier PXD017973.
