# Supplementary material for: The expression of the acarbose biosynthesis gene cluster in Actinoplanes sp. SE50/110 is dependent on the growth phase
Source: BMC Genomics. 2020 Nov 23;21:818. doi: 10.1186/s12864-020-07194-6 (PMC7682106; doi:10.1186/s12864-020-07194-6)
Supplement: Supplementary file 1 — Additional file 1 Supplementary Figures. Includes all supplementary figures. [file 12864_2020_7194_MOESM1_ESM.docx]

**Supplementary Figures**

The file **Supplementary Figures** includes all supplementary figures.

The expression of the acarbose biosynthesis gene cluster in *Actinoplanes* sp. SE50/110 is dependent on the growth phase

Julian Droste^1^, Vera Ortseifen^2^, Lena Schaffert^1^, Marcus Persicke^1^, Susanne Schneiker-Bekel^2^, Alfred Pühler^2^, and Jörn Kalinowski^1§^

Affiliations

1 Microbial Genomics and Biotechnology,

2 Senior Research Group in Genome Research of Industrial Microorganisms,

Center for Biotechnology, Bielefeld University, Universitätsstraße 27, 33615 Bielefeld, Germany

§ Corresponding author: Prof. Dr. Jörn Kalinowski (joern@CeBiTec.Uni-Bielefeld.DE)


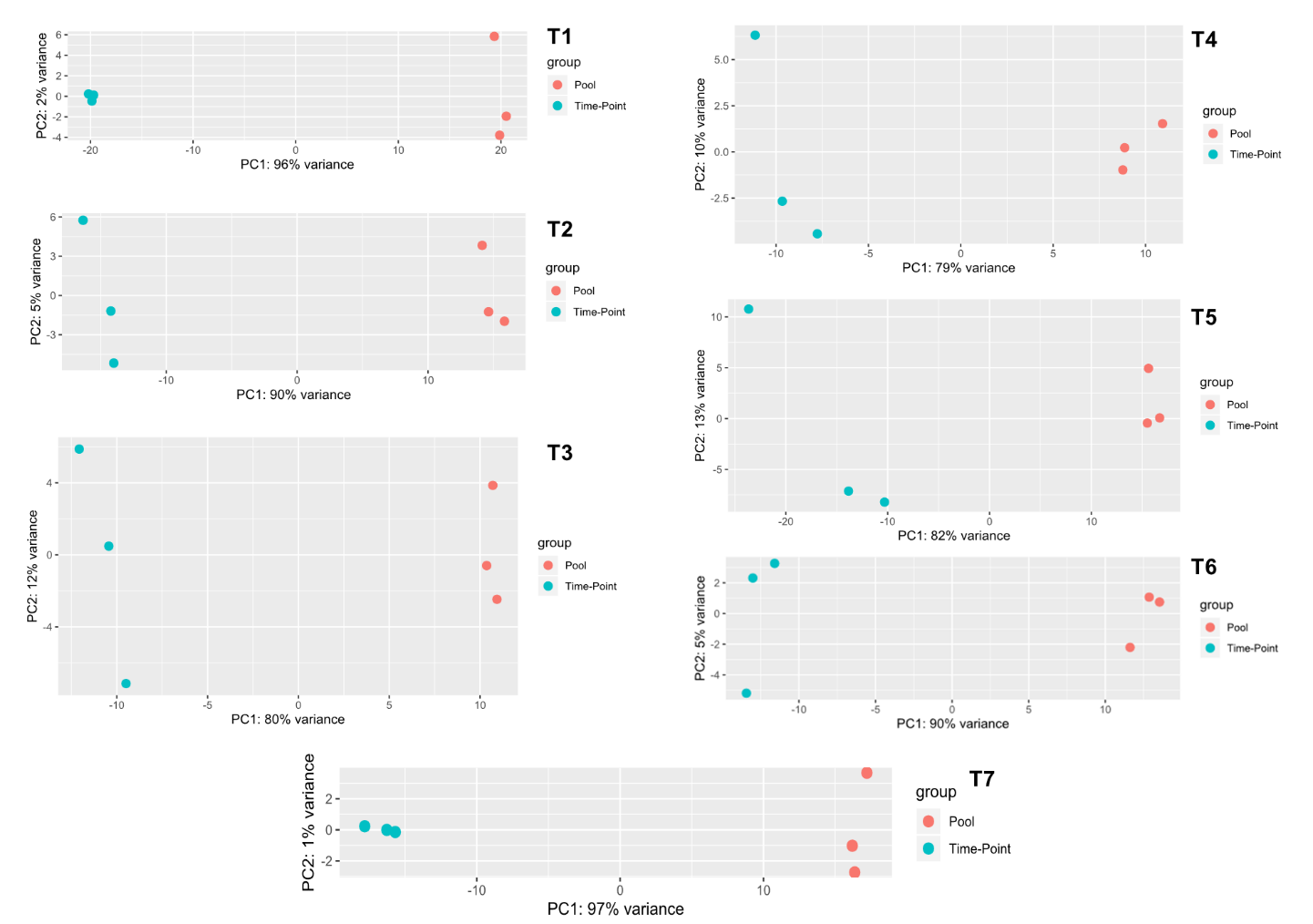


**Supplementary Figure 1:** Principal component analysis (PCA) plot of whole transcriptome data analyzed by DESeq2 [89] of an Actinoplanes sp. SE50/110 fermenter cultivation grown in maltose minimal medium in three biological replicates. Samples were taken at seven different time points (every 24 h). Each time point was compared to a pooled sample over all time points for each fermenter. The plots were generated using R (version 3.6.2).

.


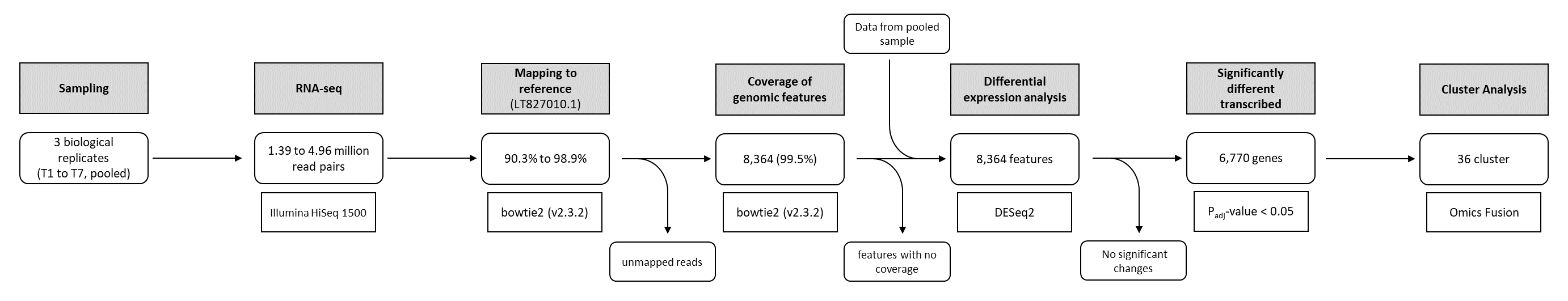


**Supplementary Figure 2:** Processing and filtering steps of the whole transcriptome analysis of Actinoplanes sp. SE50/110.

**Supplementary Figure 3:** Number of monocistronic genes, primary operons and sub-operons of *Actinoplanes* sp. SE50/110. The number of genes included in primary and sub-operons is color-coded.

**Supplementary Figure 4:** Length distribution of the 5´-untranslated regions (5´‑UTRs) based on 4,228 identified TSSs in the genome of Actinoplanes sp. SE50/110. Shown on the y axis is the absolute number of leader sequences detected for the given length interval, while on the x axis the length of leader sequences in 5 nucleotides per interval is plotted. The red bar represents leaderless transcripts with a leader sequence length of 0 to 3 nucleotides.


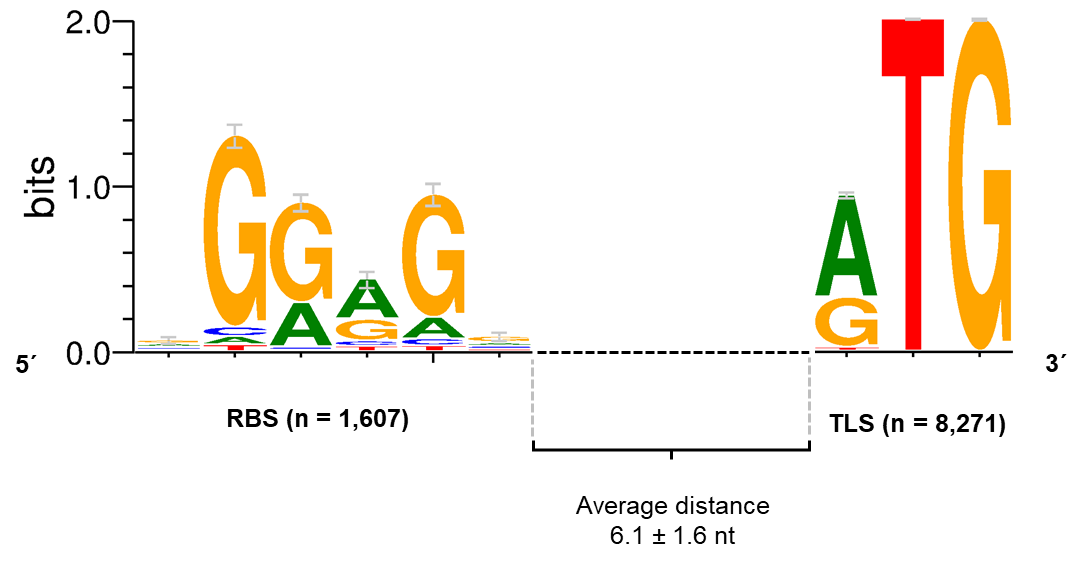


**Supplementary Figure 5:** Conserved ribosomal binding site (RBS) motif and translation start (TLS) codon of Actinoplanes sp. SE50/110. The RBS consensus sequence nGGAGn was found in 1,607 sequences upstream of the TLS of 1,725 CDSs with mapped primary TSSs and a 5´-UTR of 10-500 nt. The main TLS condons were found to be ATG and GTG. The average distance between the RBS and the TLS was found to be 6.1 nt. The conserved ribosomal binding site motif was identified using Improbizer [32], the logos were created using WebLogo (version 3.7.4) [35].


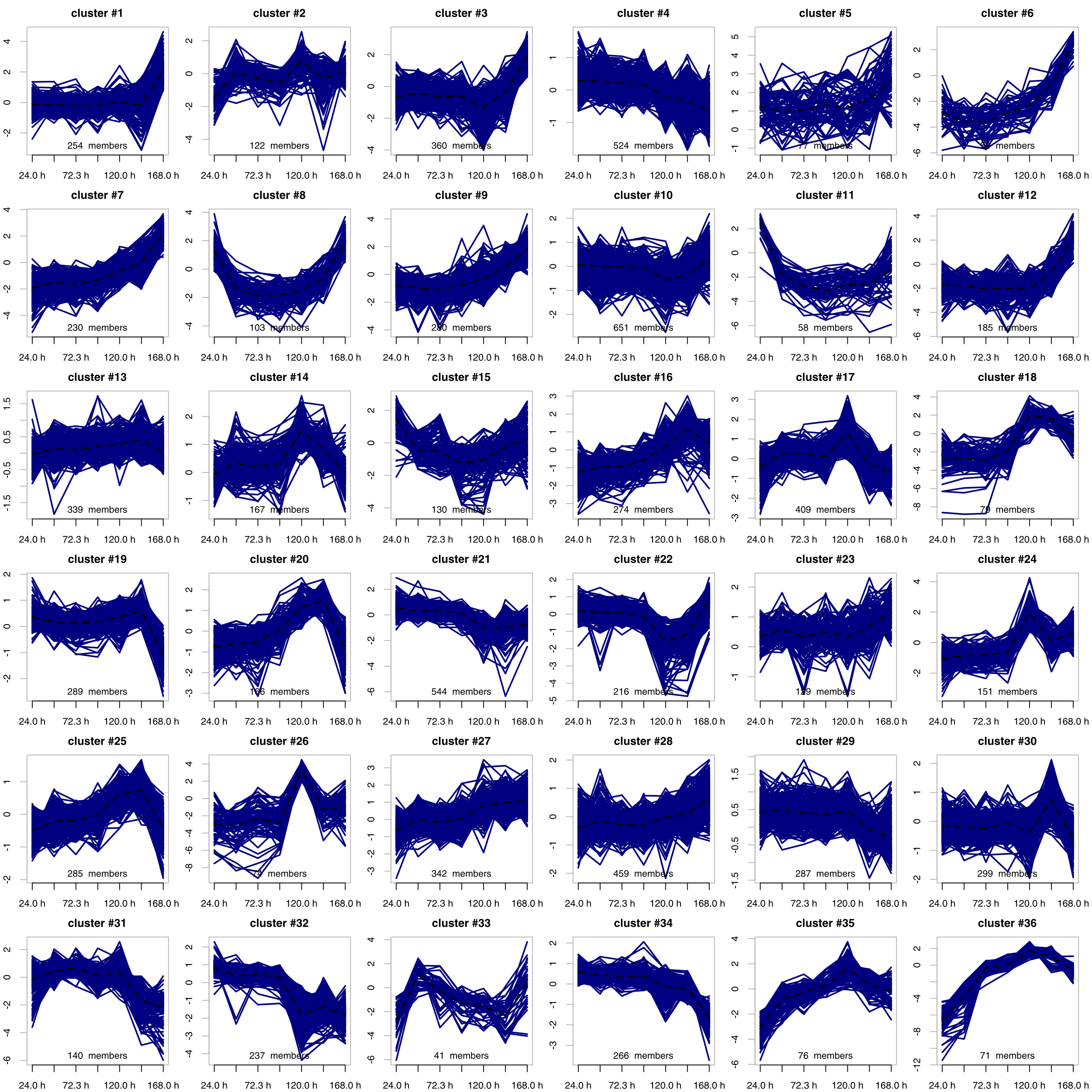


**Supplementary Figure 6:** Hierarchical Cluster Analysis of the transcriptomic data. The course of the respective M-values (log_2_ (fold-change)) is visualized for each gene of the respective cluster (blue lines). The mean value for each cluster is indicated by a dashed black line. An M-value of zero is equivalent to the pooled transcript amount over all time points (average transcript amount). The scaling of each cluster has been optimized and is therefore different. The number of genes of each cluster is shown below the respective curves. The hierarchical cluster analysis was performed using the tool Omics Fusion [36].


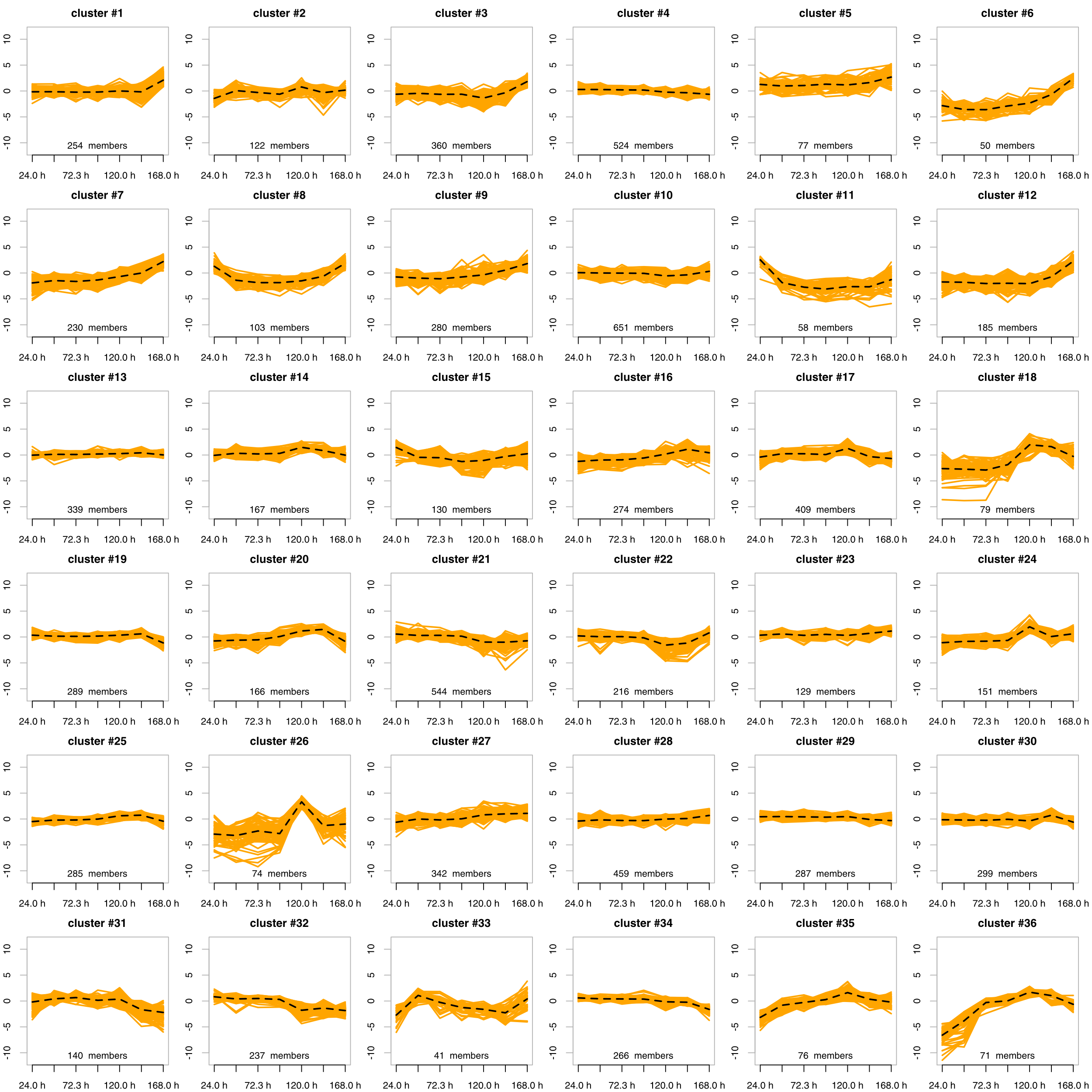


**Supplementary Figure7:** Hierarchical Cluster Analysis of the transcriptomic data. The course of the respective M-values (log_2_ (fold-change)) is visualized for each gene of the respective cluster (orange lines). The mean value for each cluster is indicated by a dashed black line. An M-value of zero is equivalent to the pooled transcript amount over all time points (average transcript amount). The same scaling was chosen for all clusters. The number of genes of each cluster is shown below the respective curves. The hierarchical cluster analysis was performed using the tool Omics Fusion [36].


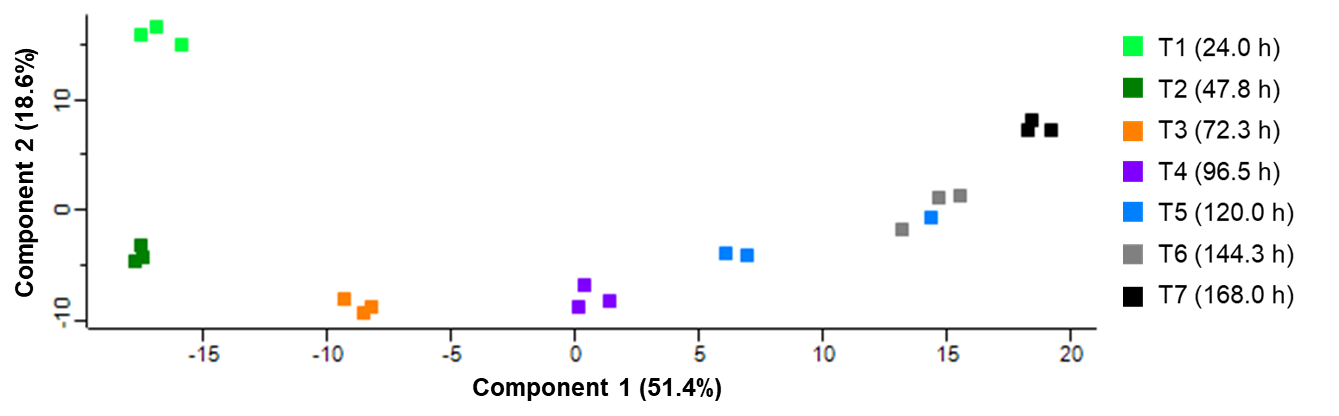


**Supplementary Figure 8:** Principal component analysis (PCA) plot of proteomic data obtained from cellular proteome of an Actinoplanes sp. SE50/110 cultivation grown in maltose minimal medium in three biological replicates. Samples were taken at seven different time points (every 24 h). The principal component analysis was performed using Perseus (version 1.6.10.43) [92].


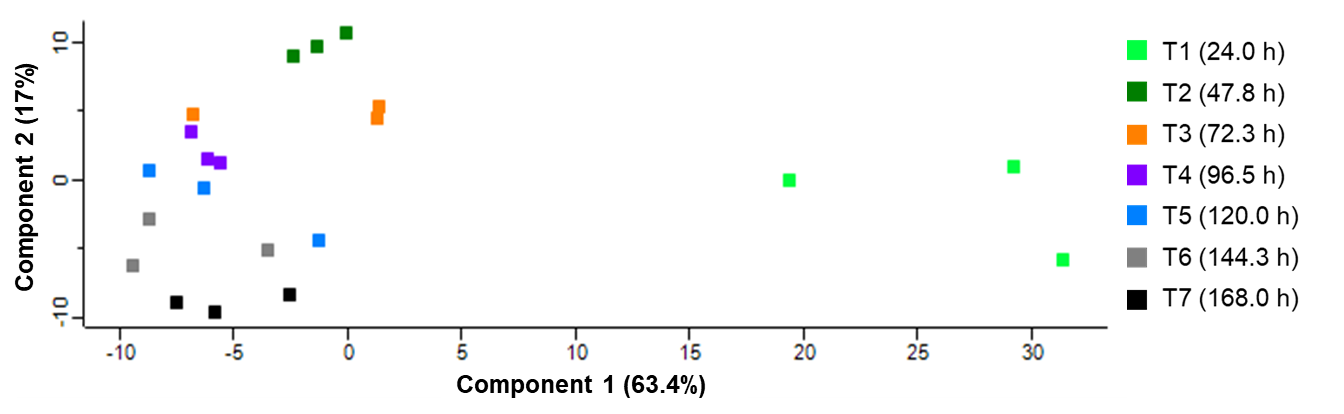


**Supplementary Figure 9:** Principal component analysis (PCA) plot of proteomic data obtained from extracellular proteome of an Actinoplanes sp. SE50/110 fermenter cultivation grown in maltose minimal medium in three biological replicates. Samples were taken at seven different time points (every 24 h). The principal component analysis was performed using Perseus (version 1.6.10.43) [92].


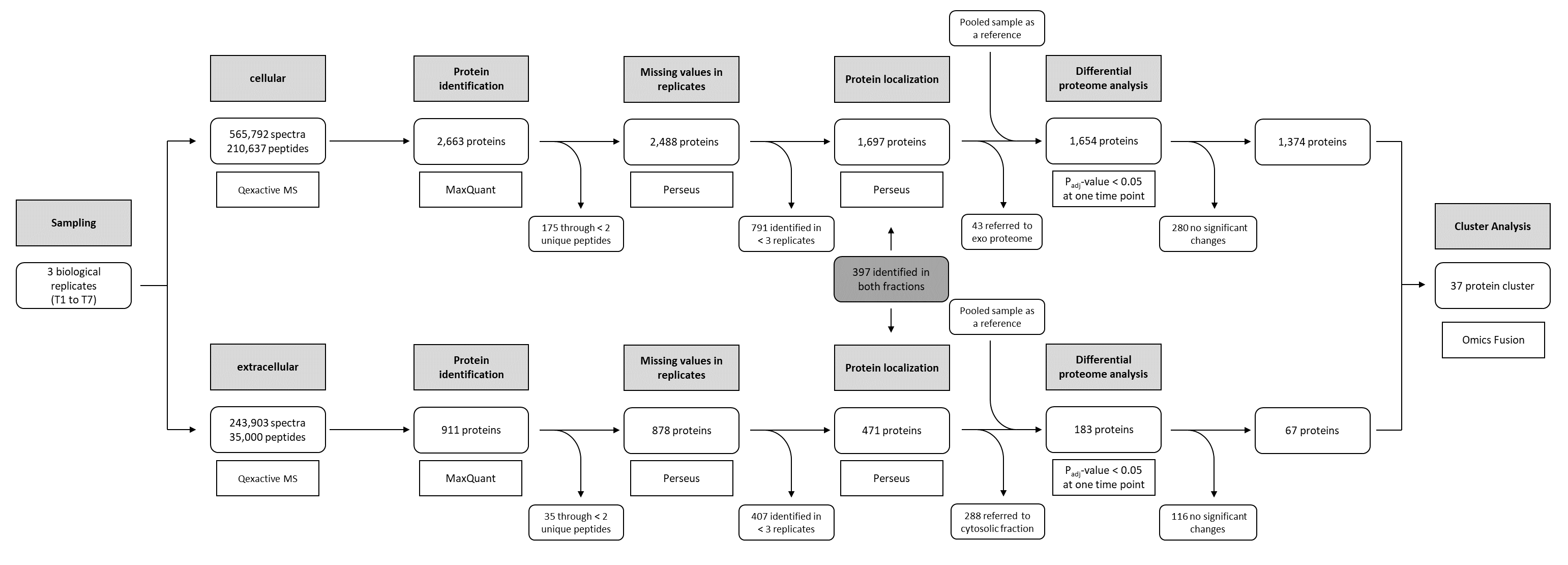


**Supplementary Figure 10:** Workflow of processing and filtering steps of the whole proteome analysis of SE50/110.


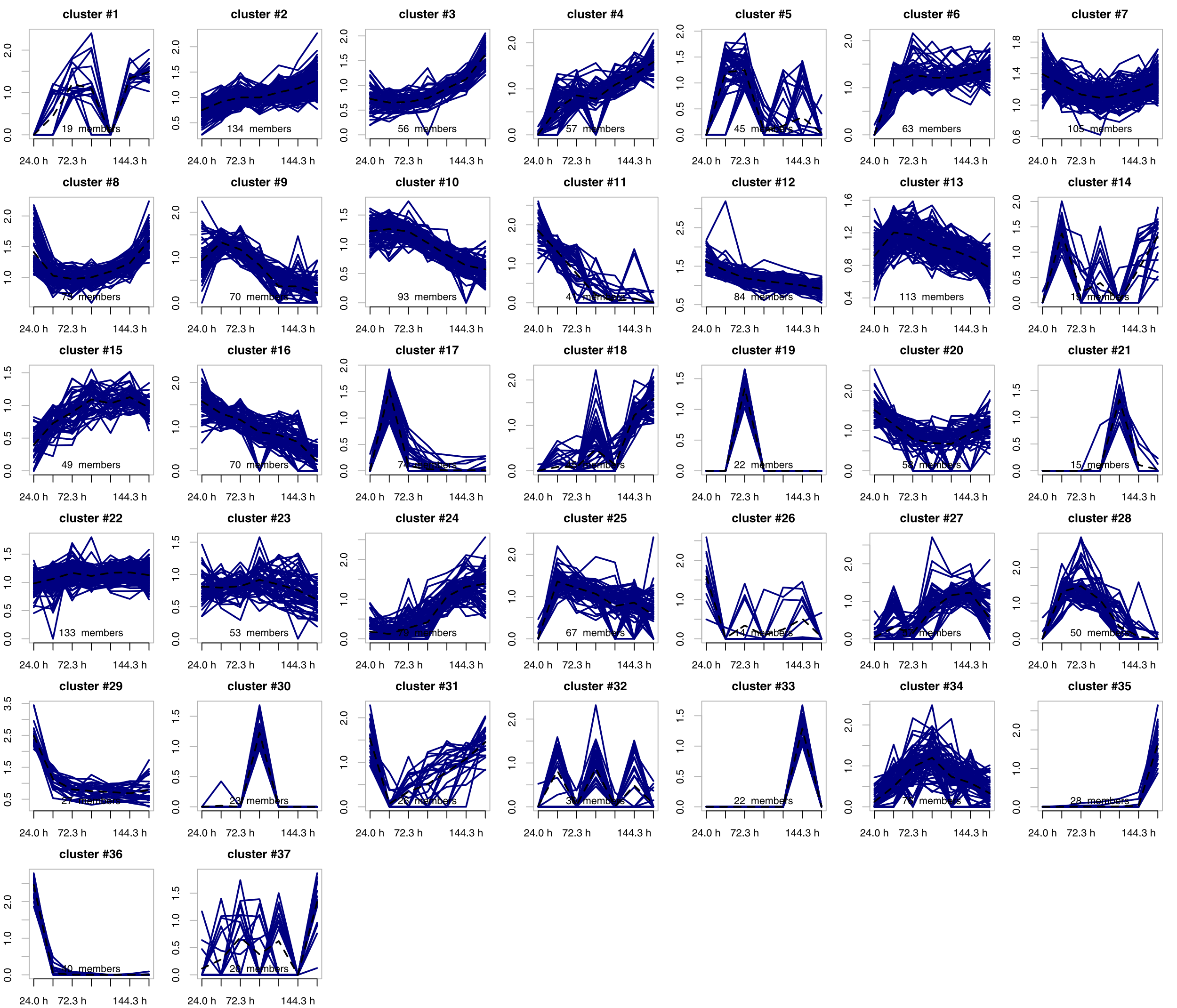


**Supplementary Figure 11:** Hierarchical Cluster Analysis of the proteomic data. The course of the respective fold-change is visualized for each protein of the respective cluster (blue lines). The mean value for each cluster is indicated by a dashed black line. A fold-change of one is equivalent to the pooled protein amount over all time points (average protein amount), whereas a value of zero indicates no available data for the protein at the respective time point. The scaling of each cluster has been optimized and is therefore different. The number of proteins of each cluster is shown below the respective curves. The hierarchical cluster analysis was performed using the tool Omics Fusion [36].


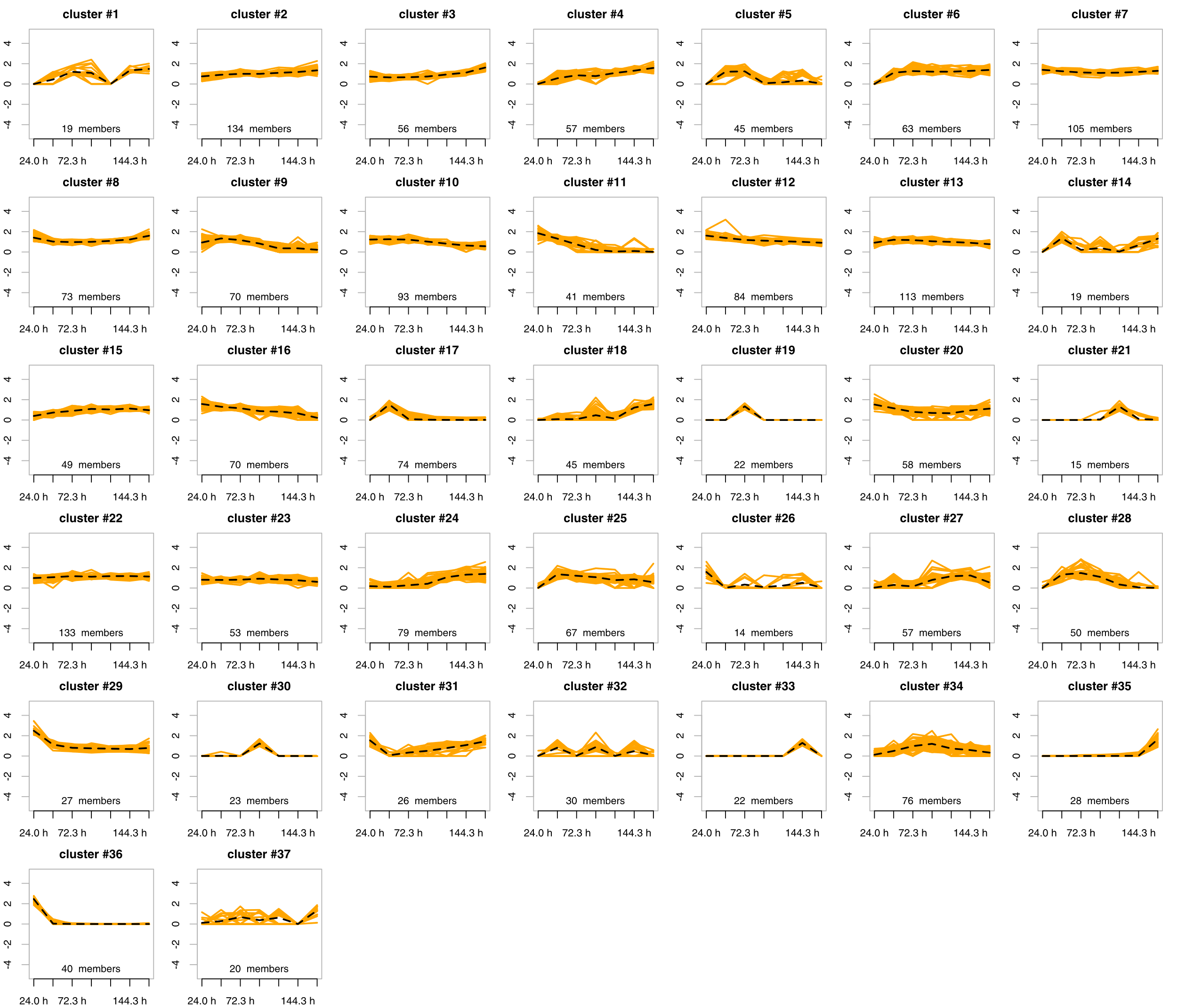


**Supplementary Figure 12:** Hierarchical Cluster Analysis of the proteomic data. The course of the respective fold-change is visualized for each protein of the respective cluster (orange lines). The mean value for each cluster is indicated by a dashed black line. A fold-change of one is equivalent to the pooled protein amount over all time points (average protein amount), whereas a value of zero indicates no available data for the protein at the respective time point. The same scaling was chosen for all clusters. The number of proteins of each cluster is shown below the respective curves. The hierarchical cluster analysis was performed using the tool Omics Fusion [36].
